# Lipid Nanoparticles: An Effective Tool to Improve the Bioavailability of Nutraceuticals

**DOI:** 10.3390/ijms242115764

**Published:** 2023-10-30

**Authors:** Rabia Ashfaq, Akhtar Rasul, Sajid Asghar, Anita Kovács, Szilvia Berkó, Mária Budai-Szűcs

**Affiliations:** 1 Institute of Pharmaceutical Technology and Regulatory Affairs, Faculty of Pharmacy, University of Szeged, Eötvös u. 6, H-6720 Szeged, Hungary; medication79@yahoo.com (R.A.);; 2 Faculty of Pharmaceutical Sciences, Government College University Faisalabad, Faisalabad 38000, Pakistan; akhtar.rasul@gcuf.edu.pk (A.R.); sajidasghar@gcuf.edu.pk (S.A.)

**Keywords:** phytochemicals, dietary supplements, functional food, SLN, NLC, liposome, nanoemulsion, herbs and spices

## Abstract

Nano-range bioactive colloidal carrier systems are envisaged to overcome the challenges associated with treatments of numerous diseases. Lipid nanoparticles (LNPs), one of the extensively investigated drug delivery systems, not only improve pharmacokinetic parameters, transportation, and chemical stability of encapsulated compounds but also provide efficient targeting and reduce the risk of toxicity. Over the last decades, nature-derived polyphenols, vitamins, antioxidants, dietary supplements, and herbs have received more attention due to their remarkable biological and pharmacological health and medical benefits. However, their poor aqueous solubility, compromised stability, insufficient absorption, and accelerated elimination impede research in the nutraceutical sector. Owing to the possibilities offered by various LNPs, their ability to accommodate both hydrophilic and hydrophobic molecules and the availability of various preparation methods suitable for sensitive molecules, loading natural fragile molecules into LNPs offers a promising solution. The primary objective of this work is to explore the synergy between nature and nanotechnology, encompassing a wide range of research aimed at encapsulating natural therapeutic molecules within LNPs.

## 1. Introduction

Nutraceuticals, natural substances that could be found in food or as part of food, are considered to be effective in terms of increasing the nutritional value of diets. They provide specific nutrients to malnourished populations, avoiding adverse effects of conventional therapy, and contribute to the prevention of certain ailments. The term ‘Nutraceutical’, conceived from Pharmaceutics and Nutrition, was introduced by Stephen Defelice in 1989 and has transformed the concept of traditional nutrition [1]. Nutraceuticals cover a great variety of substances, including phytochemicals, herbs, spices, functional foods, and dietary supplements [2]. Nutraceuticals have been classified in more than one way based on their chemical nature, source, properties, and/or disease interventions. A single nutraceutical may fall into more than one category; for example, vitamins are classified as functional foods, but some of them may also be considered dietary supplements on account of their antioxidant properties. These pharmaceutically dispensed bioactives do not solely fall in the food or medicine categories, but an area in between these two domains that is receiving a good share of attention from health practitioners [3].

The nutraceutical industry is flourishing at an incredible rate, offering a wide range of therapeutics against cancer, diabetes, hypertension, hypercholesterolemia, anxiety, depression, insomnia, analgesia, fever, cold, and cough [4]. Nonetheless, the number of processed natural ingredients is limited due to their poor solubility, low stability, and high degradation. Additionally, they often exhibit low bioavailability due to incomplete gastric absorption, excessive first-pass metabolism, and rapid clearance; thus, they face the challenges of erratic pharmacokinetic (PK) profiles [5], which are the main hurdles to deliver and achieve the desired therapeutic outcomes of bioactive natural agents. In order to exploit the maximum therapeutic potential of these natural ingredients, scientists are exploring novel encapsulation methods [6].

Recently, nanotechnology has been successfully employed in the pharmaceutical industry for improving drug solubility, bioavailability, and stability during processing and storage [7]. Nanopreparations based on lipids (LNPs), which are naturally abundant and part of the human diet, have been found to be superior to polymeric nanoparticles in preserving and enhancing the biological activity of the encapsulated payloads [8]. Low production cost, ease of scale-up, better stability profile, and ease of modifications make LNPs a more preferable option among colloidal carriers [9]. LNPs are feasible and effective for administration via different routes, including oral, parenteral, and topical [10].

Solid lipid nanoparticles (SLNs), a primary type of LNPs, are made up of solid lipids with a crystalline nature, which encapsulate the active substance within a solid lipid matrix, allowing for controlled drug release [11]. SLNs can easily penetrate through biological membranes and facilitate the penetration of loaded drugs, hence improving their concentration in target tissues [12]. Nanostructured lipid carriers (NLCs), introduced as the next generation LNPs, are composed of solid and liquid lipids. They aim to overcome the shortcomings of SLNs, for instance, increased drug loading capacity and improved stability by preventing drug leakage from the nanoparticles during storage [13].

Liposomes, nanosized lipid bilayer vesicular systems composed of natural phospholipids and cholesterol, can incorporate higher loads of both hydrophilic and hydrophobic drugs, protect the loaded substances, and efficiently cross biological membranes [14,15]. Moreover, liposomes can be easily modified for targeted delivery with controlled release and have minimal intrinsic toxicity due to the natural phospholipids. Structurally, liposomes may have one or multiple bilayer membranes. On the basis of number of bilayers, liposomes are classified as multilamellar vesicles (MLV) and unilamellar vesicles (ULV). ULV are further divided into three types: giant unilamellar vesicles (GUV), large unilamellar vesicles (LUV), and small unilamellar vesicles (SUV). ULV is comprised of a single bilayer sphere, while MLV is a structure of concentric phospholipid spheres with multiple layers enclosing the aqueous medium. Vesicular size and number of bilayers together effect the drug encapsulation efficiency [16].

Nanoemulsions (NE) or microemulsions (ME) are well-established lipid based colloidal nanoformulations stabilized with a mixture of surfactants to maintain the stability of immiscible phases (oil and water) in a colloidal state. These are mainly of three types: oil in water (o/w), water in oil (w/o), and multiphase [17]. NEs possess several advantages, including enhanced solubility, increased bioavailability, and bioaccessibility of poorly soluble drugs and nutraceuticals, as well as straightforward easy manufacturing procedure and steps. NE provide protection to natural and synthetic active compounds from adverse storage conditions, degradation, light, pH, and elevated temperature [18].

To mitigate the adverse effects of therapeutics and reduce the length of treatment procedures, nanomedicines containing bioactive materials are being launched in the global pharmaceutical market. Products that incorporate natural compounds driven by nanotechnology present better pharmacoeconomic options in both therapeutic and diagnostic fields [19]. The global nutraceuticals market size was USD 540 billion in 2022 and is estimated to reach USD 1025 billion by 2030. While the nanomedicine market size was USD 377 billion in 2021, it is projected to exceed USD 964 billion by the end of 2030 [20,21,22]. 

Our review profoundly covers recent developments in the use of LNPs for encapsulating natural active ingredients for remedial, food fortification, and diagnostic purposes. 

We are discussing SLN, NLC, liposomes, and NE as examples of lipid nanocarriers to load natural compounds in the main text. The most widely and extensively used compounds have been detailed in this review, while the less commonly used or newly established natural drugs have been summarized into tables. Special attention was also paid to formulation methods, characterization, and advantages in delivering nutraceuticals via LNPs.

**Figure 1:**
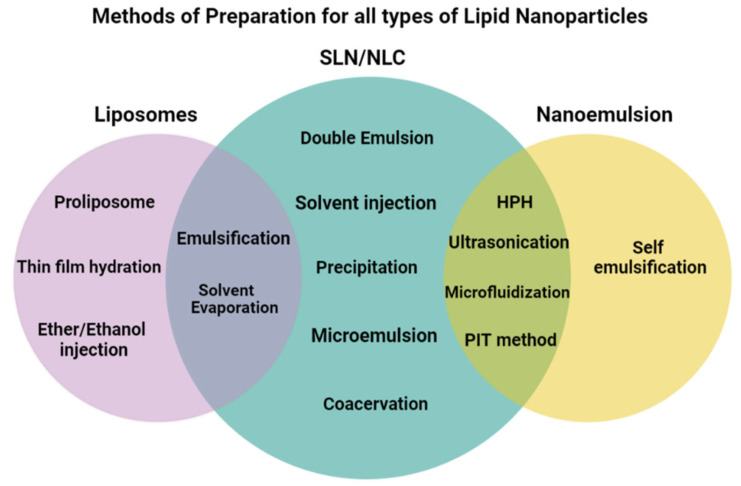
Methods of preparation for LNPs.

Lipid nanoparticles are prepared by different methods, including high-pressure homogenization, ultrasonication, emulsification, evaporation, hydration, and both high and low-energy methods. Every method has its own advantages and disadvantages that assist researchers in selecting the most appropriate option accordingly. Different methods of preparation for NLC, SLN, liposomes, and NE have been enlisted in Figure 1, while their advantages and disadvantages of each method are presented in Table 1. Moreover, a brief description of the most commonly used preparation methods has also been included in this article.

### 1.1. Methods of Preparation for SLN and NLC

#### 1.1.1. High-Pressure Homogenization (HPH)

High-pressure homogenization (HPH) is a well-established and recognized technique used for producing SLN and NLC. Lippacher et al. prepared nanoemulsions for parenteral administration using the HPH method, with the loaded drug enclosed at 5–10 °C above the melting temperature of the lipid used [23]. In this technique, high pressure (100–2000 bar) is applied to force the liquid through a very narrow gap that eventually produces high velocity (up to 1000 km/h or even more). As a result of elevated shear stress and cavitation force, the particle size is reduced to submicron level [24]. When HPH is performed at high temperatures, it is termed hot homogenization, whereas at lower temperatures, it is known as cold homogenization. Hot HPH is not suitable for thermolabile drugs or materials. In case of cold HPH, an aqueous phase is mixed with lipid phase at 2 °C to 6 °C with constant stirring. The resultant coarse suspension is subjected to HPH maintained at lower temperature. Due to the absence of elevated temperature, cold HPH is a suitable method for heat sensitive materials.

#### 1.1.2. High Shear Homogenization and Ultrasonication

Two of the most familiar dispersing techniques are high shear homogenization and ultrasonication. A warm aqueous phase containing surfactant is combined with the melted lipid under high shear homogenization, then the obtained emulsion is subjected to ultrasonication as a subsequent step to reduce the particle size to submicron level. High speed mixing and ultrasonication at relatively elevated temperature results in a smooth formulation. Intense shear forces during the ultrasonication help to break the particles to nanosize. Moreover, the type and ratio of surfactant and lipids as well as time and speed of sonication play a crucial role to produce a stable nanoformulation [25].

#### 1.1.3. Microemulsion Technique

The microemulsion technique is a multi-step method in which a surfactant solution and melted lipid are gently mixed to produce a kinetically stable dispersion.

In order to prepare a thermodynamically stable o/w microemulsion, the resultant emulsion is dispersed into sufficient amount of cold water upon continuous mixing. The ratio of water to microemulsion usually varies from 10:1 to 50:1. This dilution helps to scale down the micro formulation to nano formulation. Size of the obtained nanoparticles is dependent on two factors; one is temperature difference between cold water and hot microemulsion and the other is primary size of emulsion. NLC and SLN dispersions having micro and nano size range with spherical shapes and narrow size distribution are prepared by this method. The notable drawback associated with this method is the final diluted dispersion [26].

#### 1.1.4. Phase Inversion Temperature (PIT) Technique

The conversion of an o/w emulsion to a w/o emulsion is termed ‘phase inversion’ and depends on a change in temperature. Polyoxyethylated surfactants present different properties at different temperatures; for example, the hydration of the hydrophilic portion of the surfactant at 25 °C and the dehydration of epoxy groups occurs as the temperature increases. The temperature point at which the surfactant acquires equal affinity for both hydrophilic and lipophilic phases is known as phase inversion temperature. As the temperature continues to increase, the surfactant exhibits a greater affinity for the lipid phase, leading to a conversion into a w/o emulsion type. Weerapol prepared SLN by phase-inversion temperature method to incorporate rosemary oil (RMO) [27,28].

#### 1.1.5. Double Emulsion Technique

The most suitable method for the incorporation of peptides and hydrophilic compounds is the double emulsion technique. Initially, the drug is solubilized in an aqueous solution, which is then emulsified with lipid to form a w/o emulsion with the incorporation of suitable surfactants. The obtained w/o emulsion is dispersed in an emulsified aqueous solution to form a double w/o/w emulsion through continuous stirring. After filtration, the nanoparticles are separated. This technique tends to relatively produced large particles [29].

#### 1.1.6. Solvent Injection Technique

A fast and simple method that uses organic solvents (such as methanol and acetone) to dissolve lipids, which are then injected into an emulsified aqueous phase through a needle. The quick and gradual diffusion of lipids into the aqueous phase produces small sized droplets that could be controlled by adjusting the injection speed [30].

#### 1.1.7. Solvent Evaporation Method

Lipophilic contents are dissolved in an organic solvent and incorporated into an aqueous phase containing surface active agents. Upon evaporation, the lipid precipitates, resulting in the formation of nanoparticles with a size range of 25 nm. In the next step, the solution is emulsified by high-pressure homogenization with the removal of the organic solvent under reduced pressure (40–60 bar) [31].

#### 1.1.8. Precipitation Method

An organic solvent is required in the precipitation method; this is a drawback of this technique. In the initial step, an organic solvent (such as chloroform) and glyceride are mixed together with the subsequent addition of emulsified aqueous phase. The resultant mixture is subjected to evaporation, forming nanoparticles with lipid precipitation [25].

#### 1.1.9. Coacervation Method

The coacervation method begins with the heating of the polymer in water. Subsequently, lipids are incorporated into the hot solution while stirring continuously above the Kraft point of the lipid.

At first, the active ingredient solution is added to the resultant mixture, and finally, the coacervating solution is added dropwise to set the desired pH. Once the desired pH is acquired, the solution is cooled down to 15 °C in a water bath while mixing [32].

### 1.2. Methods of Preparation for Liposomes

#### 1.2.1. Thin-Film Method

One of the most widely used methods for liposomal preparation is the thin film hydration method. A thin film of lipid with active ingredients is formed at the bottom of a rotary evaporator and is subsequently hydrated with buffer or water. To obtain a smoother bilayer, both the lipid film and hydrating solutions should be heated above the transitional temperature of lipids. Sonication helps in peeling off the film from the surface and in the formation of liposomes. This method is used to prepare MLV (multilamellar vesicles) liposomes; it is a reproducible method but has the shortcoming of low encapsulation efficiency [33].

#### 1.2.2. Proliposome Method

This is the simplest method to prepare a larger quantity of liposomes. Compared to the thin film method, it has less reproducibility but offers higher entrapment efficiency. The aqueous solution of lipid and ethanol is heated on stirring for 10 min at 60 °C. Once the smooth lipid melt has cooled down to a lower temperature, the buffer or aqueous phase is incorporated dropwise while stirring. The obtained suspension is further hydrated for an hour to acquire MLV liposomes [34].

#### 1.2.3. Injection Methods

A lipid suspension of organic solvents is injected into an aqueous phase with some variations to prepare liposomes in larger quantities. Ethanol and ether injection methods are types of injection methods that produce SUVs (small unilamellar vesicles) and LUVs (large unilamellar vesicles), respectively. In ethanol injection method, lipids are dissolved in ethanol and injected into aqueous phase upon continuous stirring with subsequent removal of solvent. Ethanol removal from the system can be performed by centrifugation or rotary evaporation. The resultant solution is subjected to hydration afterwards. This method may lead to poor encapsulation of hydrophilic drugs. On the other hand, as ether does not mix with water and due to having high lipid solubility aspect, the ether injection method produces liposomes with increased encapsulation efficiency [35].

#### 1.2.4. Emulsification Method

The emulsification method is somewhat similar to the injection methods. According to this technique, lipids are solubilized in organic solvents and gradually mixed with the aqueous phase. An emulsion of water in oil is formulated with a lipid monolayer around the water droplet. Once the solvent is evaporated, it results in liposome formation. This method ensures higher encapsulation efficiency compared to the injection method [36].

### 1.3. Methods of Preparation of Nanoemulsion

Various high-energy methods, utilizing significant fluid stress, are employed to break down the dispersed phase into small-sized droplets when formulating nanoemulsions. Ultrasonication, high-shear mixing, high-pressure homogenization, and microfluidics are the techniques to obtain particles with controlled size and stability [37]. Gharibzahedi et al. reported on the effectiveness of high-energy methods in encapsulating nutritional food components while preserving their activation and storage [38].

#### 1.3.1. High-Energy Methods

##### High-Pressure Homogenization

HPH is responsible for reducing coarse emulsion particles to an extremely low size with the application of turbulence, hydraulic force, and cavitation during the homogenization. Other parameters that define the size of primary emulsion droplets are intensity, time, temperature, and the type of homogenization method. The application of the HPH method has been extended to pharmaceuticals, nutraceuticals, food, and biotechnological active ingredients [39]. HPH technique has been discussed in detail in Section 1.1.1.

##### Microfluidization

This high-energy mixing technique involves the use of a microfluidizer under high pressure (500~20,000 psi) to homogenize a coarse emulsion. It is a combination of imprinting technology, water jet technology, and the homogenization technique with the utilization of an interaction chamber. The process efficiency is dependent on diameter, pressure, treatment temperature, and design of the flow channel. Coarse emulsion occurs under pneumatc pressure, directed to the interaction chamber of microfludizer where it enters into narrow channels. There, emulsion streams collide with each other at high velocity, resulting in the formulation of a nanoemulsion. At the outlet of the microfluidizer interaction chamber, an in-line heat exchanger is used to cool down the resultant dispersion. Due to the fixed geometry of the microfludizer, this method showed better reproducibility [40].

##### Ultrasonication

Ultrasonication has gained more recognition compared to other high-energy techniques due to the cleanliness of operation. Ultrasonic waves are responsible for generating cavitation force to break macroemulsion into nanoemulsion. Moreover, the collapsing of microbubbles produces turbulence to break microemulsion into nanoemulsion [41]. Detailed discussion is available in Section 1.1.2.

#### 1.3.2. Low-Energy Methods

Low-energy emulsification methods are believed to be an energy-efficient method as they utilize the internal system energy and gentle stirring to produce emulsions at the nano level.

Phase inversion emulsification and self-emulsification are more common methods for producing food-grade nanoemulsions among low-energy techniques. One important point of consideration is the need for a relatively higher amount of surfactant in case of low-energy methods [42].

A spontaneous change in temperature or composition resulted in a phase change during the emulsification. Namely, phase inversion composition and phase inversion temperature are two types of phase inversion emulsification methods [43].

#### 1.3.3. Self-Nanoemulsification Method

The spontaneous emulsification method is termed the self-emulsification method. Such a system contains more hydrophilic content than lipophilic content. They behave like isotropic mixtures of lipids, drugs, and surfactants. Upon dilution in biological fluids, they form clear o/w emulsions with the help of the agitated movement of the stomach and intestinal motility [44].

### 1.4. Characterization of LNPs

**Figure 2:**
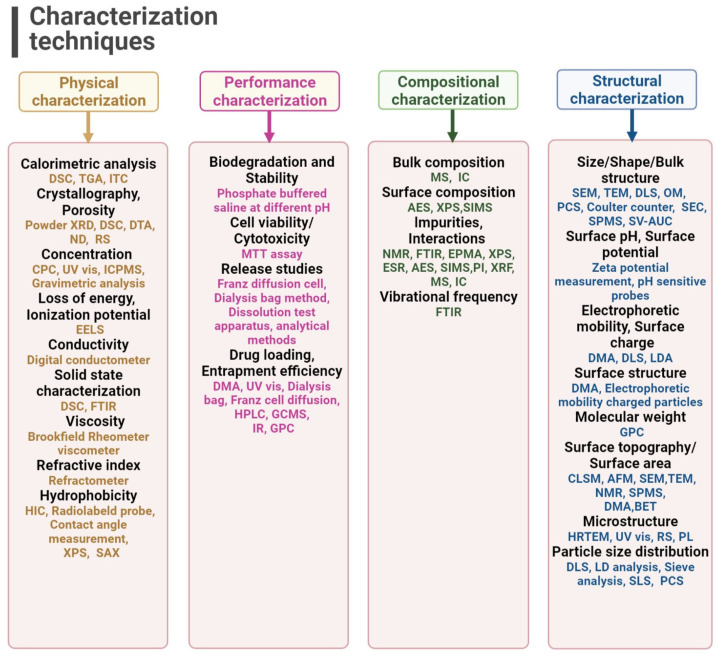
Characterization techniques related to nanoformulations. (Abbreviations: DSC, differential scanning calorimetry; TGA, thermogravimetric analysis; ITC, isothermal titration calorimetry; XRD, X-ray powder diffraction; DTA, differential thermal analysis; ND, neutron diffraction; RS, Raman spectroscopy; CPC, condensation particle counter; UV vis, ultraviolet and visible absorption spectroscopy; ICPMS, inductively coupled plasma mass spectrometry; EELS, electron energy loss spectroscopy; FTIR, Fourier transform infrared spectroscopy; HIC, hydrobhobic interaction chromatography; XPS, X-ray photoelectron spectroscopy; SAX, synchrotron radiation X-ray; MTT assay, (3-[4,5-dimethylthiazol-2-yl]-2,5 diphenyl tetrazolium bromide) assay; DMA, differential mobility analyzer; HPLC, high performance liquid chromatography; GCMS, gas chromatography mass spectrometry; IR, infrared spectroscopy; GPC, gel permeation chromatography; MS, mass spectroscopy; IC, ion chromatography; AES, atomic emission spectroscopy; SIMS, secondary ion mass spectroscopy; NMR, nuclear magnetic resonance; EPMA, electron probe micro-analyzer; ESR, electron spin resonance; PI, polydispersity index; XRF, X-ray fluorescence spectroscopy; OM, optical microscopy; PCS, photon corelation spectroscopy; SEC, size exclusion chromatography; SEM, scanning electron microscopy; TEM, transmission electron microscopy; DLS, dynamic light scattering; SV-AUC, sedimentation velocity analytical ultracentrifugation; LDA, laser doppler anemometry; CLSM, confocal laser scanning microscopy; AFM, atomic force microscopy; SPMS, single particle mass spectrometry; BET, Brunauer–Emmett–Teller analysis; HRTEM, high resolution transmission electron microscopy; SLS, static light scattering; PL, photoluminescence spectroscopy; LD analysis, laser diffraction analysis.).

During the formulation and drug development process, the physical and chemical properties of ingredients are required to be investigated. These properties are extensively studied to evaluate the stability, impact, and performance of the product. To characterize lipid nanoparticle formulations, various old and new analytical techniques have been introduced [45] and applied in the pharmaceutical sector. A comprehensive overview of these investigations is available in Figure 2.

## 2. Phytochemicals

Naturally occurring chemicals in plants that are not required for their growth but protect them from infections and diseases are termed phytochemicals. Countless phytochemicals exist in nature, and most of them have been reported for their medicinal effects, including their antibacterial, anti-inflammatory, hormonal, and enzymatic stimulation properties. According to available and published data, more than 5000 phytochemicals are known so far that are present in vegetables, vegetable oils, fruits, whole grains, and nuts [46].

In this review article, we are describing the beneficial and healthcare roles of phytochemicals in controlling and treating diseases. Moreover, we are highlighting the issues faced in the efficient delivery of phytochemicals along with practical solutions and strategies to overcome these shortcomings. In current times, lipid nanoformulations loaded with phytochemicals are receiving attention as a promising and emerging delivery systems. Most extensively used phytochemicals have been described in the text below, while the recent developments in this field are documented in Table 2.

### 2.1. Lycopene

Lycopene, a terpenoid present in watermelon, grapefruit, and tomato, is considered one of the most potent naturally occurring antioxidants [89]. It also possesses anti-inflammatory, anti-tumor, and anti-mutagenic properties [90]. In spite of its persuasive medicinal characteristics, the therapeutic role of lycopene is limited due to its low aqueous solubility and poor stability [91].

Riangjanapatee et al. evaluated the behavior of different surfactants on the stability of lycopene-loaded NLCs composed of orange wax (90% *w*/*w*) and a lycopene oil solution in rosemary oil (10% *w*/*w*). Different surface active agents, C-1216 (sucrose laurate), C-1816 (sucrose stearate), C-1616 (sucrose palmitate), C-1815 (sucrose stearate), and Plantacare 1200 (lauryl glucoside), were investigated for their ability to stabilize the NLCs prepared by hot high-pressure homogenization (HPH). It was found that Plantacare 1200 produced the smallest NLCs of all, attributed to the smaller contact angle of the surfactant with the orange wax. Both the particle size and zeta potential of the lycopene-NLCs remained constant over a period of one month. Furthermore, the chemical stability of lycopene was enhanced many folds in the NLCs (t_1/2_ = 192.5 h) compared to its solution (t_1/2_ = 9.6 h) [92].

Riangjanapatee et al. also evaluated the effect of cholesterol on the chemical stability of lycopene in NLCs. The presence of cholesterol resulted in significantly smaller NLCs but also caused the degradation of lycopene [93]. On the other hand, NLCs produced with rice bran oil significantly improved the stability of lycopene within the NLCs, and increased antioxidant activity was observed for the Lycopene-NLCs when compared to blank rice oil NLCs [94].

Zardini et al. used a combined high shear homogenization and ultrasonication technique to formulate lycopene-loaded NLCs and SLNs to evaluate the potential of nanocarriers for food fortification. The size range of nanoparticles was 74.93–183.40 nm. NLCs were found to exhibit higher encapsulation efficiency when compared to SLNs, while particle size was smaller. Nanoencapsulation of lycopene not only precluded the poor solubility but also masked its aftertaste [95]. Nanosized liposomal formulation was loaded with lycopene by Zhao and fellows to improve neuronal protection against ischemic brain injury. Neurological score, oxidative stress markers, neuronal apoptosis, and infarction volume indicated an increase in the amount of lycopene at the site of action due to encapsulation in nano-liposomes [96].

Similarly, a methotrexate-induced kidney dysfunction model was used to test the activity of lycopene in two pharmaceutical forms, one encapsulated in nanoliposomes and the other dissolved in corn oil. During in vivo studies, nanoliposome-encapsulated lycopene reported a reduction in animal kidney dysfunction condition, with a higher degree of recovery [97]. In both in vitro cytotoxicity and in vivo antitumor studies conducted on B16 melanoma-bearing mice, the combination of doxorubicin and lycopene-loaded liposomes exhibited enhanced therapeutic efficacy and reduced cardiotoxicity [98].

Li et al. used medium-chain triglycerides and octenyl succinate anhydride (OSA)-modified starch as stabilizers to create lycopene-loaded oil-in-water NE. Increased lateral packing of the stabilizer at the interface contributed to a reduction in particle size and resulted in a physically stable NE [99]. To acquire stable lycopene NE, different oil phases, such as sesame oil, linseed oil, or walnut oil, were used, with lactoferrin serving as the emulsifying agent to prepare lycopene-loaded NE. The results indicated that lycopene NE formulated with sesame oil exhibited greater stability and a slower degradation rate compared to other formulations, making it an effective choice for lycopene-fortified delivery systems [100].

### 2.2. β-Carotenoid

Tetraterpenoids are a subclass of carotenoids found in chloroplasts, fungi, and bacteria [101]. They play a meaningful role in photosynthesis by absorbing sunlight and protecting chlorophyll from damage caused by sunlight [102]. β-carotene is a commonly used carotenoid in food and health sciences and is believed to be highly active among the other carotenoids. It is known for its antioxidant, anticancer, and immune-boosting properties due to its ability to scavenge reactive oxygen species (ROS) [103]. Aqueous insolubility and low bioavailability are significant obstacles to the pharmaceutical applications of β-carotene for consumers. Different potential carriers are under investigation for the incorporation of this lipophilic compound [104].

Incorporation of β-carotene in NLCs to enhance β-carotene’s stability was performed by Hentschel et al. It was found that β-carotene degrades quickly on dilution of the dispersion. Protection of β-carotene from degradation seemed to increase after the incorporation of Vitamin E along with β-carotene. The antioxidant property of tocopherol protected the nutraceutical from oxidative, preserving its stability for 11 days in a diluted formulation and 19 days in a pure formulation. The NLCs remained stable without sedimentation and aggregation for a period of 7 months, making them suitable for use in beverages [105].

During the processing of carotenoids, physical and thermal stress decrease the stability and promote oxidative degradation [106]. LNP’s solid core may act as a shelter for carotene, protecting it against oxidation. Helgason et al. investigated the dependence of the stability of bioactive components on surfactant type, lipid crystal structure, and evaluated the activity of lecithin as antioxidant and stabilizer for the system. Crystalline structures of the lipids were achieved during the processing stages with the use of high-melting surfactants. High-melting (HM) surfactants, such as HM-lecithin, were proven to be superior to Tween 60 and Tween 80 in stabilizing the solid lipid carrier system and protecting the payload chemically. The selection of surfactants was identified as a critical factor not only in the development of lipid crystal structures but also in maintaining their integrity [107].

Yi et al. revealed that the type and concentration of the proteins affected the stability, cytotoxicity, and cellular uptake of the β-carotene. Sodium caseinate and whey protein isolate could also produce nanoparticles smaller than 100 nm. SLNs remained stable for 30 days when stored at 4 °C. Sodium caseinate was discovered to establish better physical and chemical stability in SLN systems than soy protein and whey protein isolates. The presence of phosphoserine and the ability of sodium caseinate to form a thick layer at the oil-water interface at higher concentrations contribute to the production of more stable colloidal dispersions. Furthermore, all the proteins boosted the cellular uptake of β-carotene SLNs in Caco-2 cells compared to free β-carotene [108].

Orally administered carotenoid NE stabilized with Tween 40 showed better antioxidative and liver protective effects in Wistar rats than the conventional emulsion, likely due to the smaller droplet size. Tween 40 alone was found to be more toxic to the liver than in its emulsion form, indicating that a careful investigation and analysis are recommended to select a definite amount of surfactant to design an emulsion [109].

Whey protein, gum Arabic, and soy lecithin were tested for the fabrication of a stable oil-in-water NE loaded with carotenoid (paprika oleoresin). Whey protein and soy lecithin showed better results in the context of a smaller droplet size (<150 nm) and interfacial tension, which ultimately contributed to the improved stability of the carotenoid NE [110]. In a recent study, carotenoid NE produced a prominent antiproliferative effect in breast cancer cells (MCF-7) and tumor-bearing mice by virtue of considerable reduction in epidermal growth factor (EGF) and vascular endothelial growth factor (VEGF) levels compared to the carotenoid extract [111].

### 2.3. Eugenol

Eugenol (Eu) is an active phenolic constituent of clove oil. Its antifungal activity was revealed by Chami et al. while performing experiments on rats with candidiasis (oral, vaginal) [112]. After exposure to candidiasis infection, monocytes release interleukins (IL), leading to inflammation. Eu was found to be effective in controlling the production of PG-E2, IL, and tumor necrosis factor (TNF), ultimately reducing the inflammation [113].

Eu loaded SLNs were prepared by Garg et al. to treat oral candidiasis and to improve its antifungal activity. They elaborated the results, highlighting the increased stability and therapeutic effects offered by the SLN system. Two formulations, SLN1 (caprylic triglyceride) and SLN2 (binary lipids: caprylic triglyceride, stearic acid), exhibited identical encapsulation efficiency. An increase in solid lipid concentration resulted in larger particle size. Formulation SLN2, which contains a liquid lipid (stearic acid), exhibited smaller particle size and faster drug release [114].

An NLC-gel formulation containing Eu was developed to achieve sustained drug release into dentinal tubules to treat hypersensitivity problems in gums. Initial burst release followed by slower release of drug was noted. It was claimed to improve the effectiveness of the therapy by providing a rapid relief through the initial release and then maintaining the effect for an extended period of time through the prolonged release of the bioactive from the gel [115].

Loading of Eu inside SLN may lead to increased local supply and decreased systemic absorption of the active ingredient when used for topical applications. However, the lower viscosity of SLNs can be problematic for epidermal application of creams. Garg et al. prepared Eu-SLN and incorporated them into a hydrogel stabilized by Carbopol. In vivo and in vitro testing confirmed the effectiveness of antifungal therapy with augmented occlusive properties [116]. An enhanced antimicrobial effect of the combination of ofloxacin and Eu in chitosan-coated SLNs was reported by Rodenak-Kladniew et al. Controlled drug release and enhanced formulation stability allowed superior inhibition of the growth of *Pseudomonas aeruginosa* and *Staphylococcus aureus* [117].

Hyaluronic acid (HA)-coated liposomes loaded with Dacarbazine and Eu were formulated and optimized by the Quality-by-Design (QbD) approach. Migration and proliferation assays confirmed a higher inhibitory effect against metastasis even at reduced doses [118]. Cationic and temperature-sensitive liposomes encapsulating Eu were coated onto negatively charged silk through electrostatic interactions, with the aim of achieving sustained and controlled release of fragrance from the fragrant silk. The purpose of the following study was to lower the release rate and increase the fragrance lasting time. According to release study data, lower than 30% of the fragrance was released from eugenol-loaded liposomes [119].

Eu-loaded liposomes expedited the wound closure and healing process when tested using an in vitro scratch assay wound model of neuronal and microglia origin cell lines. This observation included assessments of ROS production, cell viability, and polarization properties [120]. Chitosan-coated liposomes or chitosomes displayed enhanced Eu incorporation capacity and boosted antioxidant activity when compared to the conventional liposomes [121].

Tanzeem et al. designed a clove oil-based ME loaded with flurbiprofen to utilize the gastroprotective effect of Eu in clove oil and reduce the NSAIDs associated gastric damage. The ME system increased the solubility and dissolution of flurbiprofen, which could increase the drug bioavailability. The stomachs of the rats treated with ME showed fewer lesions and less harm to the gastric mucosa when compared to the pure NSAID in PEG, thus making it a suitable vehicle for delivering NSAIDs that may cause gastritis [122].

Fu et al. developed a stable, Eu-loaded antimicrobial NE by high-speed shearing technique, which was found to be effective against *Escherichia coli* and *Staphylococcus aureus*. Severe deformation and membrane rupture were observed in both bacterial strains through SEM, along with measurements of ROS and MDA levels [60].

### 2.4. Curcumin

The golden spice turmeric, scientifically known as ‘Curcuma longa’, is the natural source of curcumin (Cur; 1,7-bis(4-hydroxy-3-methoxyphenyl)-1,6-heptadiene-3,5-dione) [123]. Cur is frequently used in Central and South Asia as a condiment. It also acts as an iron chelator to control iron accumulation in cardiac and hepatic cells, as well as various parts of the brain [124].

Preclinical studies have been conducted by scientists for many years to investigate the therapeutic potential of Cur, compelling them to proceed with clinical trials for a proper evaluation of its effectiveness [125]. However, while Cur is considered a miracle for mankind, its poor aqueous solubility, low bioavailability, and stability problems hinder its maximum therapeutic potential.

Tiyaboonchai et al. prepared SLN with a mean particle size of approximately 450 nm. They observed the dependence of particle size and Cur encapsulation on the overall percentage of lipids and surfactants used. SLNs were successfully incorporated into a cream formulation and showed a sustained release pattern after topical administration. Stability studies exposed the ability of the SLN to improve the stability of Cur over a period of 6 months [126].

Pliangbangchang et al. studied the anti-aging effects of the Cur-SLN-loaded cream on 33 volunteers for 8 weeks. The cream was found to be safe for the volunteers, with no reports of irritation. Skin wrinkles were markedly reduced, and the hydration level of the skin also improved with the treatment. Additionally, skin elasticity and firmness also improved over the course of the testing period [127].

Kakkar et al. successfully prepared SLNs with an average particle size of 134.6 nm and a drug content loading of 92%. Pharmacokinetic studies were performed with Wistar rat plasma to investigate any changes in the bioavailability (BA) of Cur. A validated LC-MS characterization method confirmed a 39-fold increase in the bioavailability (BA) of curcumin (Cur) at a dose of 50 mg/kg and a 155-fold increase at a dose of 1 mg/kg [128].

Researchers evaluated the ability to mitigate AlCl_3_-induced neurotoxicity, which is found to be fatal for the central nervous system (CNS) functionality and could lead to Alzheimer’s disease (AD). Cerebral histopathology not only confirmed protection against neurotoxicity but also demonstrated its reversal when treated with Cur-SLN [129]. They demonstrated increased cytotoxicity of Cur-SLN against human alveolar adenocarcinoma (A549 cells), human promyelocytic leukemia (HL-60 cells), and human prostatic small cell carcinoma (PC3 cells) than the Cur solution [130]. Moreover, they also studied the protective role of Cur-SLN in cerebrovascular ischemia-induced rats and found that cognition in rats was almost restored. The Cur-SLN formulation also significantly controlled acetylcholinesterase release while improving the supply of Cur to the brain [131].

Cur also acts as a p300-HAT inhibitor, a subtype of HAT (histone acetyltransferase) enzyme that contributes to its anticancer and anti-inflammatory properties [132].

Puglia et al. conducted in vivo studies in mice, discovering that intraperitoneally administered Precirol 5 ATO and Miglyol-based NLCs, stabilized with Lutrol F68 and Tween 80, effectively improved the anti-tumor effect of Cur in CNS. Western blotting analysis of the spinal cord fluid showed revealed a decrease in the acetylation of histone (H4) at lysine (K12) when compared to the DMSO-Cur solution, which was related to better CNS permeation of the NLCs [133].

Meng et al. probed the effect of Tween 80 (PS 80) on the brain permeation of the Cur-loaded NLCs. PS 80-NLCs remained stable in the plasma and allowed the adsorption of protein that was hypothesized to improve the formulation penetration by promoting the binding of PS 80-NLCs to receptors overexpressed at the blood-brain barrier (BBB). Ex vivo imaging of mouse brain sections confirmed an enhanced BBB penetration by PS 80-NLCs (up to 2-fold) in comparison to ordinary NLCs [134].

Meng et al. designed an NLC carrier system modified with lactoferrin (Lf) to mimic low-density lipoproteins (LDLs) since their receptors (LDLR) are highly expressed in the BBB. This modification enhances the protection provided by Cur against the progression of Alzheimer’s disease in the brain. Lf-NLCs were made with phosphatidylcholine, cholesterol oleate, and glycerol trioleate stabilized with S100-COOH (Carboxylated polyethylene glycol (100) monostearate) to resemble the hydrophobic lipid core of LDL (cholesteryl esters with triacylglycerides, having a surface coat of phospholipids, unesterified cholesterol). Lf was electrostatically adsorbed onto the NLCs to match the presence of the apolipoprotein B100 molecule on natural LDLs. The results indicated that LDL-mimic-NLCs remained stable as they passed through brain endothelial cells. Imaging and histopathological investigations of rat brain sections made it clear that Cur-Lf-mNLCs had better penetration and accumulation in rat brain cells compared to lactoferrin-free NLCs They also conveyed a superior healing effect on the damaged brain cells [135].

Ban et al. used tristearin and PEG as emulsifying agents to formulate Cur-loaded SLNs for enhance oral bioavailability. The digestion of lipids resulted in the phenomenon of micellization, enabling the solubilization of a large amount of Cur. The size and charge of these mixed micelles affected the extent of absorption through the intestinal epithelium which in turn, determined the plasma level of curcumin. PEG-stabilized SLNs improved oral bioavailability in rats due to the permissible size range and neutral charge of the micelles, as well as the extended lipolysis of SLNs [136].

Singh et al. designed a heat-sensitive system by encapsulating Cur into gold-coated liposomes for the stimuli-responsive destabilization of liposomes and controlled release of Cur at the desired site. They used the B16F10 melanoma cell line to study in vitro photothermal effect and intracellular uptake. Upon laser irradiation, curcumin-loaded liposome gold nanoparticles showed enhancement in cancer cell cytotoxicity against B16F10 melanoma cells, confirming the potential of nanoliposome formulation as a potential system for photothermal therapy [137].

Cur-loaded liposomes, formulated using bovine milk phospholipids and krill phospholipids, exhibited high stability with enhanced bioavailability, as well as antioxidative and anti-hyperglycemic properties [138]. To treat infections induced by methicillin-resistant *S. aureus* (MRSA), Bhatia et al. formulated a berberine-curcumin co-encapsulation liposomal system to evaluate the synergistic antimicrobial effect of both phytochemicals. In comparison with clindamycin, co-encapsulated liposomes showed a 77% reduction in intracellular infection [139]. The co-encapsulation of curcumin and tetrandrine in nano-liposomes (CT–DP–Lip), stabilized with DSPE–MPEG 2000 (DP), yielded formulation of sizes under 100 nm. MTT studies on MDA–MB–231, HepG2, HGC–27, and HCT116 cell lines confirmed the strong anti-tumor effect of the CT–DP–Lip formulation [140].

Paez-Hernandez et al. observed that the selection of oil is crucial in the design of Cur-loaded NEs. Grapeseed oil and olive oil induced recrystallization of the Cur, whereas medium-chain triglycerides produced the most stable NE with the smallest globule size. Time and power of ultrasonication was also negatively correlated to the size of the NE. Similarly, an increase in cycles and pressure in microfluidization led to small-sized NE. However, ultrasonication was superior to the microfluidization technique in obtaining uniformly dispersed smaller formulations [141].

Ahmad et al. used central composite design to prepare Cur NE and evaluate its anti-inflammatory and wound healing effect. Clove oil, Tween-80, and PEG-400 were selected due to their higher Cur solubility. The optimized NE showed negative surface charge with a globule size below 100 nm and spherical morphology, making it appropriate for a non-toxic transdermal delivery system in wound healing applications [142]. Chuacharoen et al. studied the effect of surfactant concentration on the properties of Cur-loaded NE and realized that higher surfactant levels produced small-sized formulations but could also affect the stability under different storage and usage conditions. Nonetheless, too much surfactant caused a prominent color change in fortified milk upon storage; therefore, a balanced approach is needed when selecting the surfactant levels [143].

The antitumor activity of Cur is well-documented in the literature, and several Cur-loaded LNPs for anticancer therapy are listed in Table 3.

### 2.5. Resveratrol (Rvt)

Resveratrol (Rvt), abundant in mulberries, peanuts, and grape seeds, is a potent polyphenol that possesses anti-inflammatory, antioxidant, and antibacterial properties [161]. It is also renowned for its cardioprotective and neuroprotective nature. Moreover, its activity against brain tumors, Alzheimer’s disease, and epilepsy has also been reported [162]. The limitations faced by Rvt include its meager solubility, poor stability, and low bioavailability [163]. Pandita et al. prepared stearic acid core SLN to load Rvt for the improvement of its oral bioavailability. The results highlighted an 8-fold improvement in the bioavailability of SLN in comparison to Rvt suspension, approving the suitability of the orally administered lipid-based nanocarrier system [164].

Neves et al. prepared Rvt-loaded SLNs (cetyl palmitate) and NLCs (cetyl palmitate and miglyol-812) and examined them for stability, solubility, and permeability of Rvt across intestinal epithelial cells. They found increased penetration of Rvt loaded LNPs. It was further demonstrated that oral absorption was enhanced during mealtime due to excessive secretions of intestinal juices [165]. Furthermore, SLNs were functionalized with apolipoprotein E (ApoE) to facilitate its passage more effectively through the BBB, as ApoE can bind to the overexpressed LDL receptors present on the BBB. It was discovered that the functionalized formulation successfully penetrated the brain and provided better protection and stability to the loaded entity [166].

Cur and Rvt co-loaded SLNs have also been reported to display excellent anticancer activity against colorectal cancer cells Rvt loaded liposomes were fabricated for mitochondrial-targeted delivery to overcome the narrow plasma half-life of Rvt, which hinders its permeability and retention. Researchers claimed improved targeting of therapeutics and the stimulation of the mitochondrial signaling pathway through Rvt-loaded liposomes. These liposomes were proven to be effective against multidrug-resistant cancer [167].

### 2.6. Hesperetin

Hesperetin, a flavone, is known for its powerful antioxidant and antitumorigenesis properties [168]. It has inhibitory effect against cardiac diseases and colon cancer [169]. The main obstructions for the use of hesperetin in pharmaceutical field are its poor physicochemical attributes (very low solubility, bitter taste) and erratic pharmacokinetic traits (poor dissolution and low bioavailability) [170].

Hesperetin-loaded NLCs were formulated by Fathi et al. for food fortification of milk. Glycerol monostearate and stearic acid were evaluated as solid lipids, whereas a mixture of oleic acid and estasan were used as an oil phase. Investigations revealed the NLCs of smaller size with greater encapsulation capability were obtained when compared to SLNs. Furthermore, glycerol monostearate produced smaller nanoparticles than stearic acid. Nanoencapsulation in NLCs not only improved the solubility of hesperetin but also masked its unpleasant taste. Burst release and low charge on NLCs’ surfaces were identified as the drawbacks of the formulations that need to be addressed in further research [171].

Wang et al. prepared hesperetin NE using pseudo-ternary phase diagrams and optimized it with response surface methodology. The extent of bioavailability and C_max_ showed increases of more than 5-fold and 2-fold, respectively, compared to hesperetin suspension. Hesperetin NE effectively improved lymphatic transport, intestinal permeability, and bioavailability [172].

A tumor-targeting hyaluronic acid (HA)-modified liposome, loaded with cisplatin (CDDP) and hesperetin, was intended to relieve systematic toxicity and anti-metastasis of triple-negative breast cancer. HA modification facilitated cellular uptake in MDA-MB-231 cells. Co-loaded formulation suppressed intracellular signaling pathway PI3K/Akt/mTOR, epithelial-mesenchymal transition, and tumor metastasis, while showing a greater safety profile against normal cells [173].

### 2.7. Capsaicin

Capsaicin (CAP), a lipophilic pungent tasting constituent of hot pepper, is illustrious for its ability to lower blood pressure, alleviate hyperlipidemia [174], and its widespread use in the treatment of conditions such as arthritis, neuropathy, sciatica, neuralgia, and other inflammatory conditions [175].

Administering CAP is problematic due to its low bioavailability and rapid clearance, requiring the use of organic solvents for solubilization because of its aqueous insolubility. CAP has been encapsulated in SLN gel by Sharma and Arora using the high shear homogenization-ultrasonication technique. Skin permeation studies showed 1.6 times greater penetration in comparison to unprotected CAP. It was summarized that the usage of SLN gel as a carrier potentiated the anti-inflammatory effects of CAP and also gave better and prolonged release pattern in topical administration without using organic solvent [176]. Duangjit and et al. discovered the positive effect of limonene on the topical delivery of CAP-loaded cetyl palmitate-Transcutol P SLNs. This effect was seen as an increase in the permeability and encapsulation efficiency of CAP-SLN. Penetration enhancer effect of the terpene was remarkable but its effect on the stability of the CAP in the SLN was not evaluated [177].

Kunjiappan and coworkers encapsulated capsaicin in SLNs for the treatment of human hepatocellular carcinoma. They used the solvent evaporation-emulsification technique to formulate SLNs. SLNs with an 80 nm diameter showed better aqueous stability. In vivo biodistribution studies confirmed that 15% of SLN formulation was presented in hepatic cells while 0% was found in the brain cells. As the CAP-VEGF receptor (vascular endothelial growth factor) complex in the liver was highly stable, researchers concluded that capsaicin-loaded SLNs are effective against carcinoma due to their comparatively long circulation time (up to 3 days) [178].

### 2.8. Naringenin (Nar)

Naringenin (Nar; 4′,5,7-trihydroxyflavanone), a naturally occurring flavonoid, is reported to be present in cocoa, tomatoes, fruits such as cherries, and grapefruit [179]. The structure-activity relationship of Nar has been revealed, demonstrating how its hydroxyl groups (OH) are involved in exhibiting antioxidative properties, as OH groups donate hydrogen for the reduction of ROS [180]. Nar has also been also documented for its anti-inflammatory, anticancer, and hepatoprotective properties [181]. The major shortcoming in utilizing Nar is its lower bioavailability when administered orally on account of its insoluble nature in aqueous media [182].

Ji et al. formulated Nar-loaded glycerol monostearate SLNs for pulmonary applications using an emulsification and low-temperature solidification method. They also optimized the formulation by Taguchi orthogonal design. Sustained release of Nar for up to 48 h was observed for Nar-containing SLNs. Additionally, Nar-containing SLNs were also efficiently taken up by A549 cells in a time-dependent manner. Relative pulmonary bioavailability was recorded to be two times greater, with a prolonged half-life for the SLNs when compared with the suspension of Nar [183]. Wang et al. formulated paclitaxel and Nar co-loaded SLNs with Pericol ATO5, Dynasan 114, and Lutrol F188 as the solid lipid, surfactant, and stabilizer, respectively. RGD peptide sequence (cyclic) was added through the chemical conjugation process for surface functionalization.

Drug entrapment efficiency was found to be more than 80%. Pharmacokinetic studies revealed an increased absorption rate of the active substances from the SLNs. U87MG glioma cells were used to perform cytotoxicity studies, and florescence dye uptake measurement confirmed the elevated uptake of the SLNs formulation compared to the blank solution. Co-loaded SLNs modified with cRGD showed improved cellular uptake and anticancer activity [184]. Munir et al. evaluated three types of LNPs, stearic acid (SA), stearic-lauric (SL), and lecithin-chitosan (LC), loaded with Nar in an arthritis model of albino male rats. RA factor, key inflammatory markers, and joint damage inspection showed a tremendous decrease with LNPs in contrast to pure Nar. SL-LNPs showed better anti-inflammatory and therapeutic effect of NAR, followed by LC-LNPs and SA-LNPs [185].

To address the poor solubility and bioavailability issues of Nar, Wang et al. constructed Nar-loaded metal-organic framework (MOF) and further encapsulated this inclusion complex into liposomes that showed sustained release of Nar. The novel formulation was found to be very effective against lung cancer cells (A549 cells) and gastric cancer cells (SGC-7901 cells) [186].

### 2.9. Baicalin

Baicalin (BA), an active flavonoid, extracted from *Scutellaria baicalensis*, has been reported for its anti-inflammatory, antiviral, and antioxidant properties [187]. Tu et al. described the neuroprotective activities of baicalin and its effectiveness against cerebral ischemia and reperfusion injury [188].

Liu et al. formulated a BA-loaded pegylated cationic SLN modified with OX26 antibody (OX26-PEG- CSLN) to examine the amino acid levels in rats suffering from ischemia and reperfusion disorder. The levels of excitatory amino acids (EAA) and inhibitory amino acids (IAA) in the extracellular fluids of rats were determined as indicators to monitor the effectiveness of BA-loaded OX26-PEG-CSLN. Enhanced brain penetration of OX26-PEG-CSLN was observed with superior modulation of EAA (glutamate (Glu), aspartic acid (Asp)) and IAA (glycine (Gly), taurine (Tau), and γ-aminobutyric acid (GABA)) in comparison to BA solution [189].

Zhang et al. functionalized BA-loaded liposomes with Borneol (BO) (BO-BA-LP) and evaluated it for pharmacokinetic and pharmacodynamic behavior. A significant increase in the half-life of BA was noted, resulting in a marked improvement in the neurological and pathophysiological conditions of the middle cerebral artery occlusion rat model [190].

BA-loaded lipid-based NE was manufactured to evaluate its absorption by the lymphatic system, using the chylomicron flow blocking model. The lymphatic transport ability was also measured utilizing the lymph node distribution method. Compared to the BA-containing suspension, the C_max_ value was 11.5-fold higher for the NE in the rats’ lymph nodes, presenting it as an effective treatment for chronic hepatitis B [191].

### 2.10. β-Sitosterol

Phytosterols, such as β-sitosterol, are structural components of the plasma membrane that are crucial for the developmental and regulatory processes in plants [192]. For human consumption, they are available in the market in the form of phytosterol-enriched butter, cereals, and margarine [193]. β-sitosterol is considered useful in lowering total cholesterol levels and low-density lipoprotein levels. β-sitosterol has also been found effective against cancers and ROS [194].

Lacatusu et al. designed β-sitosterol and green tea extract containing NLCs to improve antioxidant activity. The results showed that the use of green tea extract along with β-sitosterol was beneficial for attaining smaller NLCs with enhanced scavenging activity. Moreover, the use of natural oils such as grape seed oil helped in attaining a sustained release behavior of the loaded active [195].

β-sitosterol-loaded SLN, formulated by the double emulsion solvent displacement method, was assessed for its anti-arthritic effect against complete Fruend adjuvant (CFA)-induced arthritis. β-sitosterol-SLN reduced the levels of cytokines, cyclooxygenase-2 (COX-2), prostaglandin E2 (PGE2), VEGF, and nuclear factor kappa light chain enhancer of activated B cells (NF-κB) while increasing the redox status of synovium, superoxide dismutase, glutathione, and catalase, as well as the expression of HO-1 and Nrf2. Therefore, a pronounced anti-arthritic effect from β-sitosterol-SLN was reported [196].

Liposomal-encapsulated β-sitosterol (LS) was administered to mice by oral gavage to study the inhibition of colon tumor invasiveness. Cell viability, protein expression, and invasiveness were determined by MTT assay, western blotting, and invasion assay, respectively. Bioluminescent imaging and ELISA were performed to monitor tumor growth inhibition and determine biomarkers in the intestinal epithelium, respectively. LS exhibited a significant anticancer effect and fewer metastases, which were attributed to the pronounced immune stimulation indicated by elevated levels of interleukins [197].

### 2.11. Sesamol

Sesame seed or sesame oil contains lignans such as sesamolin and sesamin, which can account for up to 1.5% of the total weight. An antioxidative phenol component known as sesamol (5-hydroxy-1,3-benzodioxole or 3,4-methylenedioxyphenols) is generated through the treatment of sesame oil or the hydrolysis of sesamolin [198]. Sesamol possesses features of a molecule with good oral bioavailability according to the Lipinski rules, but it has erratic pharmacokinetic attributes [199]. Sesamol is a sparingly soluble compound with approximately 35% documented oral bioavailability in Sprague–Dawley (SD) rats [200]. Sesamol undergoes fast elimination within the first 4 h and exhibits low oral bioavailability [201].

VanGilder et al. elaborated the antioxidative properties of sesamol [202]. Additionally, sesamol has been reported to have anti-aging, cardio-, hepato-, and chemoprotective activities [203].

Kakkar et al. designed sesamol-loaded glyceryl behenate SLNs to alleviate the CNS abnormalities associated with menopause, including cognitive loss and anxiety. Sesamol-SLNs with a mean diameter of 122 nm and entrapment efficiency (EE) of about 75% were obtained. Scintigraphic images of a rat model, coupled with biochemical and behavioral assessments, proved the superiority of the oral sesamol SLN formulation as a remedy for neurodegenerative disorders [204]. The progression of liver diseases is mostly associated with the presence of ROS [205].

Sesamol acts as scavenger due to the presence of a benzodioxole group, making it a potential remedy for managing liver disease [206]. Sesamol was encapsulated into SLNs by Singh et al. to evaluate its hepatoprotective ability. Spherical nanocarriers were formulated with the use of Compritol, Tween 80, and soy lecithin via the microemulsification technique. In vivo testing was conducted on rats with CCl_4_-induced sub-chronic hepatic toxicity. The ultimate levels of liver function markers showed the significant effect of sesamol-SLNs in terms of reducing hepatotoxin levels in the blood at a low dose of 8 mg/kg, as compared to silymarin (25 mg/kg) [207].

Disease induction and recovery effects were assessed through histopathology and by monitoring the levels of liver function biomarkers. Results showed significantly improved hepatic recovery with seasmol-SLNs [208]. Sachdeva et al. administered sesamol-loaded glyceryl behenate SLNs stabilized with Tween 80/soy lecithin in rats with ICV-STZ induced memory deficits. Sesamol was found to be a highly effective neuroprotectant when delivered by SLNs, as it lowered the levels of TNF-α, which is responsible for increased inflammation, neural cell damage, and, ultimately, memory dysfunctions [209].

Skin exposure to ultraviolet light leads to the production of ROS [210] that cause damage to skin in the form of skin aging and, ultimately, skin tumors as a result of DNA mutation [211]. Endogenous antioxidants act as the first line of defense, but they may need backup. To replenish antioxidants, exogenous scavengers are applied to skin [212]. Geetha et al. evaluated the anticarcinogenic effect of sesamol SLNs cream base in mice. Enhanced retention of cream base in the skin was observed. Skin permeation studies conducted by ex vivo experiments also showed a significant retention effect of the formulation. The reversal of cell damage was observed in sesamol SLNs in the mouse models of benzopyrene-induced skin cancer [213].

W/O/W NE containing sesamol and retinol were optimized with the aid of response surface methodology for food fortification applications. A deposited layer of alginate and chitosan surrounding the droplets of NE was formed via electrostatic attraction to control the release of encapsulated components. The sustained release of both sesamol and retinol were reported in simulated gastric and intestinal media, revealing the suitability of the biopolymer functionalized nanosystem for loading multiple payloads and increasing the shelf-life of the system [214].

As a conclusion of the above section, Table 4 presents the summary of potential phytochemicals loaded in lipid nanocarriers.

## 3. Functional Foods

Functional foods are natural substances used in the diet or as part of the daily diet that can have significant positive effects on health and disease management, beyond their nutritional value. Records of various functional foods can be found in this article that were discovered through a comprehensive review of the literature.

### 3.1. Vitamin B_12_

Vitamin B_12_ (Cobalamin), an essential vitamin for living organisms, works as a coenzyme for the synthetic pathway of DNA, where it helps in the synthesis of purine and thymidine and also contributes to the methylation of DNA [245]. Cell propagation inside the body also increases the demand for cobalamin, as this process involves thymidine and methionine for growth [246]. Genetic disorders are also considered to be a consequence of cobalamin deficiency as structural defects in genetic materials occur in the absence of cobalamin, which leads to interruption of DNA methylation. All these abnormalities subsequently result in the development of cancer [247].

Researchers packed cobalamin within the Tween 80 stabilized Compritol SLNs to enhance its anticancer activity. Cobalamin-SLNs were negatively charged and much smaller (200 nm) than free cobalamin (650 nm). Cobalamin-SLNs facilitated the easy passage of cobalamin across the cell membrane with a sustained release profile, thus resulting in a higher death rate of cancerous cells compared to free cobalamin [248]. Singh and colleagues exploited the receptor-mediated transport of the cobalamin through the intestinal epithelium to improve the oral absorption of amphotericin B-loaded SLNs (VBS-AmB-SLNs). Higher stability against the digestive enzymes and increased safety against J774A.1 cells were noted for VBS-AmB-SLNs. Moreover, favorable interaction with mucin was also observed for better absorption of the SLNs. The cobalamin functionalized SLNs were preferentially taken up by macrophages and were superior to the simple drug solution in antileishmanial activity [249].

To treat various neurological disorders, coencapsulation of alpha-lipoic acid (ALA) and cyanocobalamin in NE was performed by Coban et al. Castor oil was found to be suitable for encapsulating both ingredients, and optimum NE in terms of globule size, PDI, and zeta potential were obtained by formulations made by magnetic stirring and fine-tuning of the agitation speed, temperature, and pH. NE was found to be suitable, in comparison to the solution form, for extending the stability of both encapsulated molecules along with a desirable release profile [250].

Transferrin (Tf)-functionalized cobalamin-liposomes were reported to improve the CNS penetration of cobalamin, allowing it to effectively exert its anti-amyloidogenic activity, which is beneficial for treating Alzheimer’s disease (AD). This is particularly advantageous given hydrophilicity and the higher molecular weight of cobalamin. The formulation displayed excellent physical stability, sustained cobalamin release, slowed the formation of Aβ fibrils, and degraded mature fibrils, hence proving its effectiveness against AD [251].

### 3.2. Vitamin A

Vitamin A (Retinol) is responsible for vision, healthy hair and skin, and mucous membrane production. It can also lead to the peroxidation of lipids present in skin. This fat-soluble vitamin has the ability to moisturize the skin in order to retain its freshness and elasticity. Additionally, it possesses an anti-wrinkle effect that reduces skin degeneration and keratoses. When applied topically, it can also acts as an antioxidant against the aging of the skin [252].

Jenning et al. performed release kinetics studies on retinol-loaded glyceryl behenate SLNs using Franz diffusion cells and correlated release behavior with polymorphic transitions of lipids. After the topical application of SLNs, water evaporated from the formulations and promoted the interaction of SLNs with electrolytes present on the surface of the skin. This interaction resulted in retinol expulsion due to transitions in lipid crystals. The incorporation of surfactant controlled the transition, but only for a very short time period. Using a mixture of surfactants, gelling agents, or humectants could slow down the lipid transition (upon skin contact) to the β form and provide a sustained release effect [253].

Pople et al. discovered that retinol-loaded SLNs incorporated into a gel could easily target and deliver high concentrations of retinol at the application site. Enhanced penetration of the active ingredient was prominent with improved hydration when using the SLNs-loaded gel. The concentration of retinol (determined by penetration studies) was almost double for the SLN-enriched gel compared to the conventional gel [252].

Retinol and ascorbic acid co-loaded NE prepared through microfluidization were evaluated for cytotoxicity and regulation of milk-specific protein synthesis. Changes in αs2-, β-, and κ-casein expressions were higher in NE compared to conventional vitamin administration, revealing that the vitamin-loaded formulation has the potential to influence the expression of milk-related proteins [254].

### 3.3. Vitamin C

Vitamin C (Ascorbic acid) is famous for its antioxidative properties and apoptotic effects against cancerous cells. Guney et al. incorporated ascorbic acid into SLNs through high-pressure homogenization for efficient and sustained delivery of this potent antioxidant to cancer cells. It was concluded that the SLNs proved to be an effective and potential carrier for protecting the loaded vitamin and enhancing its uptake by rat fibroblast cells compared to free ascorbic acid [255].

Jiao et al. optimized the preparation of ascorbic acid-loaded liposomes using response surface methodology, as the physicochemical properties of the nanocarriers define their biological fate and performance. The optimum liposomes, with a size of 270 nm, a zeta potential of −41 mV, and a loading content of 75%, were obtained at a pressure of 25 MPa, a temperature of 48 °C, and a vitamin to phospholipid feed ratio of 0.25. A complex interplay of independent variables was discovered that needs to be carefully optimized for an efficient delivery system [256].

Hou et al. used ascorbic acid-functionalized liposomes for transfection by loading antimicrobial peptide and cathepsin B (AMP-CatB) mRNA. They suggested that multidrug-resistant bacteria can be treated in immunocompromised mice by adoptive transfer of macrophages containing antimicrobial peptides linked to cathepsin B in the lysosomes (MACs). The results demonstrated that adoptive MAC transfer was effective due to the presence of vitamin C lipid nanoparticles, which helped accumulate AMP-CatB in macrophage lysosomes to eradicate *S. aureus* and *E. coli* [257].

A mixture of w/o NEs containing ascorbic acid and collagen, formulated with tocopherol, safflower oil, surfactant, and water, was reported to exhibit pronounced protection against skin damage caused by ultraviolet radiation. This was examined using fibroblast cell lines to assess skin permeability, healing, and cell viability. Ascorbic acid potentiated the production of collagen in the skin, which synergized with the administered collagen and resulted in superior healing properties and protection against ultraviolet B (UVB) irradiation [258].

### 3.4. Vitamin D

Vitamin D (calciferol) is a fat-soluble vitamin necessary for bone health whose deficiency leads to rickets in children (especially among African-American young people) and osteomalacia in elders [259]. Calciferol deficiency is more prominent among dark-skin people; the increased melanin level in their skin causes the hindrance in calciferol synthesis in the skin [260]. Some other contributing factors responsible for the deficiency of this important fat-soluble vitamin include limited consumption of vitamin-enriched food, clothes type, habitat, reduced exposure to sun light, and excessive use of sunscreen [261]. Naturally, calciferol is synthesized subcutaneously inside living organisms, and the kidneys are responsible for its conversion to the active form. Nonetheless, the ability to produce and activate this vitamin in the body decreases with age, obesity, and malabsorption of fatty foods [262]. Thus, the exogenous fortified supply of calciferol, such as fortified milk and fortified margarine, is crucial [263].

Patel et al. prepared tripalmitin SLNs stabilized with Tween 20 to fortify ergocalciferol (an active form of calciferol). Increasing the concentration of ergocalciferol resulted in smaller SLNs with a gradual decrease in particle size from 120 nm to 65 nm as the proportion of ergocalciferol increased from 0% to 20%. The results indicated a high loading capacity of SLNs for fortifying juices with ergocalciferol [264].

Encapsulation of cholecalciferol in liposomes improved its stability and retention in the skin. Liposomes allowed 1.65 times more retention of the vitamin within the skin than its solution form. Moreover, histopathological observations revealed that the liposomal form of the vitamin augmented the production of collagen fibers, repaired the surface morphology of the skin, and mitigated the ageing phenomenon in a rat photoaging model [265].

### 3.5. Vitamin E

Vitamin E (Tocopherol), a natural antioxidant with lipophilic nature, is used for protecting exposed skin from UVB radiation. Tocopherol, beneficial for both curing and preventing [266], has a scavenging action against free radicals that promote skin aging and wrinkling [267]. Tocopherol also inherits anticarcinogenic activity, especially against UVB-induced photocarcinogenesis [268].

Dingler et al. evaluated cetyl palmitate SLNs for stabilizing tocopherol, as it is susceptible to chemical degradation. The penetration of tocopherol was enhanced by the SLNs due to the occlusive features of the SLN-based cream. The chemical nature of tocopherol remained intact for 6 months in the formulation. Moreover, SLNs also increased the aesthetic appearance by masking the uneven color [269]. Ma et al. used UVB-irradiated HaCaT keratinocytes to evaluate the photoprotective nature of tocopherol-loaded NLCs (VE-NLCs) made by the HPH technique. The nanosize of the formulation helped to produce occlusive properties, and the MTT assay confirmed photoprotection. Moreover, increased cellular uptake of VE-NLCs by HaCaT keratinocyte was also observed [270].

An ocular formulation of dorzolamide loaded in SLNs stabilized with D- α-Tocopherol polyethylene glycol 1000 succinate (TPGS) was optimized using the Box–Behnken design. In simulated tear fluid, the SLNs exhibited burst release of the drug within the first 2 h, followed by sustained release over a period of 10 h. Compared to the solution form, 2.87 times increase in corneal permeation was noted for the SLNs. Moreover, tocopherol stabilized SLNs were found biocompatible for ocular applications [271].

A tocopherol NE co-loaded with naringenin was tested for the management of Parkinson’s disease through multiple in vivo behavioral studies (akinesia test, narrow beam test, muscular coordination, and grip test) to confirm the effectiveness of the noninvasive intranasal delivery system. A negatively charged formulation with a droplet size of 38 nm was found to achieve higher trans-nasal mucosal flux, that could avoid first-pass metabolism and systemic distribution of the payloads. Significant reversal of Parkinson’s disease was attributed to the raise in SOD and GSH levels, coupled with the reduction of MDA when the co-encapsulated formulation was administered with levodopa [272].

Tocopherol-containing unilamellar liposomes were prepared using the ethanol injection method to study the stability and release of tocopherol, aiming to develop a semen cryopreservation protection system. Release studies revealed that the liposomes retained the tocopherol for 24 h, which was extended to 48 h upon the addition of cholesterol to the system, thereby improving semen motility. Moreover, stability studies confirmed the stability of system for 12 months at 4 °C [273].

### 3.6. Vitamin K

Vitamin K, a cofactor responsible for wound healing, has anti-hemorrhagic effects and plays a significant role in atherosclerosis by carboxylating proteins [274]. It also promotes hemostasis in nourishing infants [275]. Deficiency of this important cofactor may result in bone demineralization, especially in females. Oral administration of vitamin K causes a greater reduction in the international normalized ratio (INR) compared to subcutaneous administration [276]. Pereira et al. evaluated the variability in vitamin K absorption through the intestinal membrane in young patients with cholestatic diseases and elderly individuals with liver abnormalities [277].

Işcan et al. found SLNs suitable for the topical delivery of mosquito repellent (N, N-diethyl-m-toluamide) and vitamin K in terms of particle size, zeta potential, and short-term stability. The incorporation of vitamin K did not affect the particle size and zeta potential of the SLNs. Co-encapsulated SLNs were proven to be superior in terms of short-term stability, as analyzed by DSC, photon correlation spectroscopy, and zeta analyzer [278].

Liu et al. encapsulated vitamin K in SLNs for oral administration and optimized the formulation for particle size and entrapment efficiency by screening 12 lipids and surfactants with the aid of a design of experiment. About 85% of the vitamin K was entrapped inside SLNs at a feed of 5% and remained stable for more than 50 h in simulated gastric and intestinal conditions (in vitro testing) and also for 4 months on storage at ambient room temperature, thus indicating the suitability of the SLNs for oral delivery of vitamin K [279].

### 3.7. Omega(ω)-3 Polyunsaturated Fatty Acids

Omega(ω)-3 polyunsaturated fatty acids are considered heart-friendly, as they may reduce the risk of cardiac attacks and associated mortality rates [280]. Foods enriched with ω-3 fatty acids are in high demand nowadays. Companies using fish oils and flaxseeds for this purpose face challenges related to low stability and a high risk of degradation and toxicity during processing and storage. The stability of the solid lipid carrier can be improved with the addition of polyunsaturated fatty acids. Fish oil was reported to decrease the melting point of tripalmitin, inhibit the phase transition, and ultimately enhance the stability of SLNs during storage [281].

Muchow et al. produced fish oil-loaded glyceryl tristearate NLCs with masked aroma and enhanced oral absorption characteristics [282]. High melting (HM) lecithin, when used as a surfactant, was found to prevent the oxidation of the ω-3 fish oil in the SLNs without the use of antioxidants. Salminen et al. deduced that the ability of the saturated layer of HM-lecithin to solidify at interface on cooling, before the solid (high melting) lipid of the carrier, could have induced the crystallization of the carrier lipid. This generated a shell that may prevent the oxidation of ω-3 fish oil by limiting its diffusion to the outside [283].

Extract of the microalga *Nannochloropsis gaditana,* enriched in ω-3 fatty acids (eicosapentaenoic acid and glycolipids), was loaded onto SLN to protect the labile fatty acids and to control their release. More than 60% of the extract was encapsulated within the SLNs, which remained stable over a period of one month, as indicated by DSC and rheological studies [284].

A nanoliposomal encapsulation of ω-3 fatty acids (using chia oil) and α-lipoic acid (LA) was developed by researchers to protect the payloads from oxidation. Characterization techniques, including FTIR, NMR, and DSC, confirmed the suitability of carrier system with an entrapment efficiency of almost 80%. Cow milk fortified with ω-3 fatty acids and α-lipoic acid co-loaded liposomes was shown to improve its nutritional value [285].

As a conclusion of the above section, Table 5 presents the summary of potential functional food loaded in lipid nanocarriers.

## 4. Dietary Supplements

Several enzymes, antioxidants, amino acids, and metabolites that increase the essential nutritional value of a diet are termed as dietary supplements and are considered valuable for maintaining a balanced diet. In this chapter, the most prominent ones are summarized and presented from a nanoformulation point of view.

### 4.1. Melatonin

Melatonin, also known as N-acetyl-5-methoxytryptamine, is a hormone secreted by the pineal gland. Exogenous melatonin is categorized as a dietary supplement by the FDA that regulates the circadian rhythm. Deficiency of melatonin may contribute to aging, neurological, and cardiovascular disorders [315].

Exogenous melatonin is believed to correct many circadian rhythm disorders, for instance, delayed sleep phase syndrome (DSPS) and desynchronosis [316].

It is a potent antioxidant and neutralizer of nitric oxide (NO), hydrogen peroxide (H_2_O_2_), hydroxyl (OH) free radical, singlet oxygen (O_2_), and peroxynitrite anion (ONOO^−^) [317].

Some scientists have also documented the antiapoptotic property of melatonin [318]. Melatonin also contributes to reducing cardiotoxicity induced by cyclosporine A (CsA), which has also been reported to produce hepatic and renal disorders. Rezzani and colleagues evaluated the effectiveness of melatonin to improve cardiac health in CsA-treated rats [319]. They found that melatonin-encapsulated SLNs were more effective against CsA-induced cardiomyocyte apoptosis, as SLNs boosted the cellular internalization of melatonin [320].

In order to achieve and maintain a prolonged and sustained melatonin plasma concentration, special carriers other than conventional drug delivery systems are needed [321]. Melatonin undergoes first-pass metabolism when administered orally and also shows rapid plasma clearance, resulting in low bioavailability [322].

In the case of intravenous (i.v) bolus administration, its elimination half-life appears to be 40 min [323]. An in vivo study was conducted by Priano et al. to investigate the transdermal and oral delivery of melatonin-loaded SLNs in healthy human volunteers, with the aim of establishing a sustained release system for melatonin with the desired pharmacokinetic performance. Significantly higher AUC and t_1/2_ were observed for the oral SLN formulation of melatonin, but there was a 20-min delay in T_max_ compared to the melatonin standard solution. However, the transdermal SLN preparation gave a slow absorption and elimination pattern, maintaining steady plasma levels of melatonin for up to 24 h. It was concluded that LNPs can considerably increase the absorption and plasma levels of melatonin, especially in the case of transdermal delivery [324].

Melatonin is a neurohormone which is also secreted in the eyes of mammals [325]. It is stated that the level of melatonin production varies throughout the circadian rhythm; its retinal level increases to maximum at night and decreases during the daytime [326]. Intraocular pressure (IOP) is inversely related to melatonin concentration. Pressure reaches its peak level during the day and gradually lowers to a minimum at night. This fact forms the basis of the hypothesis that melatonin does have an impact on IOP through the involvement of G-protein-coupled receptors (GPCRs) [327].

Alcantara et al. demonstrated in mice that melatonin can reduce IOP when used as medication [328]. The structure of the eye (corneal outer layer) and the presence of protective mechanisms, such as lacrimal secretions, tear production, and eye blinking, contribute to the low bioavailability of administered drugs and cannot be effectively addressed by conventional drug delivery systems [329].

With the aim of increasing the ocular residence time of melatonin, Leonardi et al. prepared bioadhesive SLNs with the help of cationic lipid didecyldimethylammonium bromide (DDAB). The positive charge on the surface of SLNs facilitated electrostatic interactions between the nanoformulation and ocular epithelial cells, which are negatively charged due to mucin. This interaction resulted in prolonged residence time and enhanced penetration of the active ingredient. In rabbits, melatonin-loaded SLNs produced a significant and sustained reduction in IOP that lasted for 24 h [330].

An elastic liposome loaded with melatonin (MLT-EL) was investigated for its efficacy against UV-induced skin photoaging. Permeation studies revealed a 1.5-fold improvement in the penetration of elastic liposomes compared to simple liposomes. Increased skin hydration, coupled with the reinforcement of collagen fibers, indicated the restoration of skin elasticity upon treatment with MLT-EL in photoaging experiments [331].

Chitosan coating was employed to fabricate melatonin-loaded mucoadhesive NE (CS-MLT-NE) to improve the bioavailability of melatonin in the brain through intranasal administration. Pharmacodynamic behavioral analysis of both the NE and suspension, performed using locomotor activity tests and the FST (forced swimming, climbing, and immobility), showed a markedly improved performance of the depressive rats treated with CS-MLT-NE. This indicates its potential as a treatment for depression [332].

### 4.2. Coenzyme Q10

Coenzyme Q10 (CoQ10), a lipophilic cofactor, is a potent antioxidant consisting of one quinine ring and a side chain of polyisoprenoid [333]. It helps to promote oxidative phosphorylation and ATP synthesis by transferring electrons in the respiratory chain. In its reduced form, CoQ10 shows potential antioxidative properties by reducing oxidative damage to genetic materials, proteins, and lipids [334].

CoQ10 exhibits more efficient free radical removal activity in the epidermal layer of the skin (10-fold higher) compared to the dermal layer [335].

CoQ10 also supports the anti-inflammatory activity of fibroblasts in the dermal layer. In the elderly, levels of CoQ10 in plasma and tissues decrease. This reduction in CoQ10 concentration is believed to be associated with skin aging and exposure to ultraviolet irradiation, necessitating compensation through external sources of CoQ10, such as anti-aging or CoQ10-rich creams. Cutanova Nanorepair Q10 cream was the first NLC cosmetic product launched in 2005 by Dr Rimpler Wedemark and contains CoQ10 for skin hydration and repaire. Pardeike et al. compared Cutanova Nanorepair Q10 cream with an NLC-free cream. In vivo results showed that the NLC contributed to enhanced skin hydration as compared to the NLC-free cream. It was evaluated that the NLC formulation was good in consistency and spreadability, which improved the aesthetic appearance of the formulation [336].

Schwarz et al. reported efficient dermal delivery of CoQ10 using ultrasmall NLCs (cetyl palmitate and dioctyl ether) with a size of 80 nm, which was more effective than the same-sized NE or the conventional NLCs (200 nm). Higher concentrations of the ultra-small NLCs were observed in vitro in the deeper layers of porcine skin [337]. The strongest antioxidant effect of the ultra-small NLCs was also evident in human keratinocyte (HaCaT) cells [338].

Phosphatidylcholine (PtdCho) and apolipoprotein (apo) A-I-containing lipid nanodiscs (NDs) were loaded with CoQ10. Oxygen consumption of the HepG2 cells significantly increased upon exposure to NDs due to the localization of CoQ10 within the mitochondria, which increased oxidative phosphorylation. Hence, the antioxidant potential of the cofactor was improved due to the designed NDs [339].

Chitosan-coated liposomal formulations encapsulating carbamazepine (CBZ) and CoQ10 were fabricated and optimized by the response surface methodology. Spherical nanoparticles with encapsulation efficiencies of over 75% for both payloads and size below 200 nm were obtained. These liposomes remained stable for 3 months and were deemed suitable for the treatment of epilepsy [340].

El-Leithy et al. evaluated the CoQ10-loaded isopropyl myristate NE stabilized with a mixture of Tween 80 and Transcutol HP for anti-aging and anti-wrinkle properties. The NE sustained the payload release (47% release in 24 h) and increased its transdermal flux, improving the smoothness of the skin and significantly decreasing the skin wrinkles [341].

## 5. Herbs and Spices

Herbs are the flowering or leafy parts of plants that are used for medicine, flavor, and aroma. Examples of herbs with antioxidant properties include oregano, sage, peppermint, lemon balm, *Cinnamomi cortex*, *Scutellariae radix*, garlic, nutmeg, cayenne, thyme, clove, and many others. Some herbs and Chinese herbal medicines that have been loaded into SLNs, NLCs, liposome, and nanoemulsion are summarized in Table 6.

*Allium sativum* (garlic) has been used widely all over the world as a food and a medicine for four millennia. It is a potential nutraceutical that has been used to treat cancer, headache, ulcers, and wounds [381]. Garlic contains a number of active constituents, such as PGs, vitamins (B, E, C), fatty acids, phospholipids, amino acids, lectin, and etc. [382]. Most of the biological functions of garlic are due to the presence of organosulphur compounds such as allicin, g-glutamylcysteines, diallyldisulphide (DADS), diallylsulphide (DAS), and numerous others [381].

These compounds are not only responsible for the medicinal properties of garlic but also its distinct flavor and aroma. Ajoene is another important constituent of garlic which remains stable in aqueous medium and can be extracted through chemical processes. Ajoene is considered to be a potent anti-carcinogenic and antithrombotic agent and has also demonstrated antifungal activity [383].

Wencui et al. worked on encapsulating garlic oil in SLNs to address the issue of volatility and low solubility associated with garlic oil. The SLNs remained stable during storage after lyophilization and exhibited minimal loss of oil, despite its volatile nature. However, PK studies in rats revealed rapid clearance of the garlic oil SLNs compared to garlic oil injection. This clearance was attributed to the enhanced phagocytosis of SLNs [384].

Faran et al. utilized the thin film hydration method to prepare nanoemulsomes containing lovastatin, along with ginger oil and garlic oil. These formulations were evaluated for their ability to lower lipid levels in a rat model fed a high-fat diet. The addition of either oil to the nanoparticles boosted the antihyperlipidemic activity of lovastatin and significantly reduced the fatty liver biomarkers. Moreover, liver function tests along with histopathological observations of the liver and kidney revealed the protective effects of these oils against the disease-induced damage [385].

Quach et al. formulated liposomes using lecithin that were functionalized with ginger (*Zingiber officinale*) oil to improve the stability of the lecithin-based liposomes. Incorporating ginger oil provided pH stability to the liposomes and added intrinsic antibacterial attributes due to its shogaol-6 content. The liposomes showed sufficient loading of the active ingredient and demonstrated pH-dependent release behavior, iterating its suitability as a potential drug delivery system [386].

## 6. Current Gaps, Challenges, and Future Directions

Nutraceuticals are not merely single natural molecules or ingredients; they consist of a complex mixture of multiple compounds. A number of active ingredients are present in one biological source, and higher doses may be require for daily administration [387]. Isolating and evaluating the biological activities of these complex structures can be challenging compared to chemical drugs with well-defined structures and documented activities. Among these bioactives, many compounds are sensitive to light, oxygen, heat, humidity, and pH. Physical and chemical instability, poor aqueous solubility, degradation, safety, preparation methods, and analytical techniques entail many challenges in the formulation of nutraceutical-based delivery systems. To formulate a stable and effective delivery system for these natural substances, a complete knowledge about their physicochemical properties, interactions with drugs and excipients, suitable formulation and characterization techniques, and storage conditions in harsh environment is needed [388].

Multiple extraction techniques, such as counter-current extraction, microwave-assisted extraction, Soxhlet extraction, maceration, and percolation, are used to obtain plant extracts. Bioactive compounds from plant extracts and concentrates are produced by a biofermentation process. Concentrates may also contain excipients for processing, like spray-dried carriers [389].

Conventional solvent extraction methods are associated with the several shortcomings, involving extensive, multiple steps, time consuming process with a need of a substantial amount of solvent. Furthermore, the most significant drawback of solvent extraction method is the loss or decomposition of thermolabile nutrients from the raw material [390].

Extraction and processing techniques could retain pathogens along with spices and raw herbs. That is why processing technology and extraction methods should be efficient in removing all contaminated materials and microbes from raw herbs and spices. Radiation exposure is practically prevailing in the food industry to disinfect herbs and eliminate contaminated pathogens and hazardous materials. This radiation exposure helps extend the shelf life of herbs by eliminating infectious agents. To professionally isolate potential active compounds, the Supercritical Fluid Extraction (SFE) technique has gained fame. SEF assists in removing pathogens, insects, and other types of contaminants from natural materials; but high maintenance costs and CO_2_ consumption are major obstacles to overcome [391]. New developments in the methods of extraction, refining, and separation are required to further improve the quality of herbs and spices.

Nutraceuticals are obtained and extracted from different natural sources, for example, food, plants, fungi, and microbes. Nutraceuticals are collected, isolated, purified, and graded through analytical procedures. The entire processing may cause degradation, chemical instability, and a loss of the quality of the active substance [392].

Furthermore, there are numerous challenges that can hinder the recovery of a quality product from natural sources, including contamination and intentional or unintentional adulteration. Unintentional adulteration could arise from several factors, such as harvesting at different plant growth stages, the quantity and quality of fertilizer, microbial attack, contamination with dust, pollens, insects, rodents, parasites, fungi, mold, toxins, harvesting means, and storage conditions. These factors, among others, can be responsible for serious illnesses, infections, and sensitive organ injuries, for instance, hepatic impairment. Standardized quality control and quality assurance procedures are mandatory to ensure the purity of raw materials and finished products. Other than unintentional adulteration, intentional adulteration is performed to gain economic benefits that could result in life-threatening issues. Addition of unlabeled and undeclared synthetic elements alters the quality and pharmacological properties of the finished products [393]. For instance, *Panax ginseng*, a traditional medicine, was found to be adulterated with the roots of *Panax quinquefolius* L. and *Eleutherococcus senticosusmaxim*, which may cause serious health issues [394].

Another example of intentional adulteration is replacing grape seed products with cheap and widely available peanut skin extract. Grape seeds contain a high content of polyphenols that are used to treat neurodegenerative and cardiac disorders. On the contrary, incorporation of peanut skin extract can pose serious allergic reactions to the body. Similarly, the leaf extract of *Ginkgo biloba*, effective against cerebral vascular insufficiency, was adulterated with free flavonols such as rutin and quercetin. Furthermore, to increase the weight of a product, vegetable oils or several other low-quality oils are added, compromising the final product quality. The presence of undeclared components could also lead to serious health problems. Therefore, comprehensive regulatory measures to control such practices are a pressing priority [395].

Due to the low concentration of active ingredients in plants, many plant sources are needed. Increasing demand will endanger plant species, as seen with *Taxus brevifolia*, which bears the potential ingredient taxol. Isolating taxol from the bark is tricky, time-consuming, and costly [396]. The need for a huge amount of medicinal plants has put the local ecological environment to serious threats of shortage of this medicinal plant. Various species of *Taxus*, particularly in China and India, have been listed as critically endangered due to high demand [397].

Hence, to meet the demand, several substandard, adulterated, and inferior quality herbal and plant supplies are hitting the market, compromising the safety and efficacy of nutraceuticals.

Quantification of a sole compound or its constituents can be challenging due to the unavailability of certain validated techniques. Various analytical methods are applied to determine the purity of obtained extracts, notably, thin-layer chromatography, ion exchange chromatography, size exclusion chromatography, high-speed counter-current chromatography, high-performance thin-layer chromatography, high-performance thin-layer chromatography-heated electrospray ionization-high-resolution mass spectroscopy, nuclear magnetic resonance imaging, Fourier-transform infrared spectroscopy, and mass spectroscopy. Issues reported in using these bioanalytical characterization methods include the need for frequent cleaning, throughout sample preparation, sensitivity of the method, accuracy, and potential interference [398].

In addition to the several other analytical issues, noteworthy considerations include the selection of isolation and sampling methods, the absence of regulatory methods, and reliable reference materials [399]. The use of transformative and less destructive sampling techniques is essential to address substandardization issues. Given the complex nature and wide physical diversity of bioactives, it is recommended to utilize a combination of several analytical techniques for ingredient quantification. The combination of methods should be in accordance with the regulatory framework. Spectroscopic methods, voltammetric methods, and chromatographic methods are the main classes of analytical procedures for evaluating nutraceuticals. Furthermore, to ensure risk management and to address safety and toxicity concerns, worldwide acceptable and approved procedures should be implemented [400].

**Figure 3:**
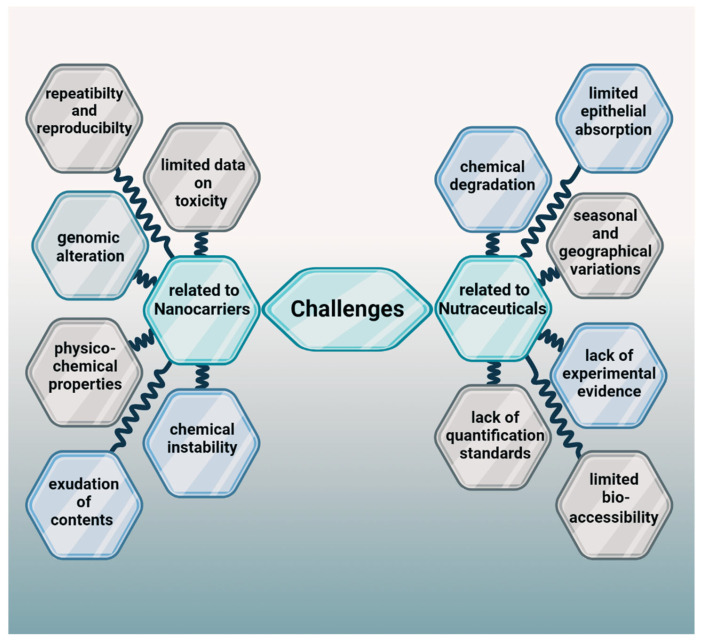
Challenges associated with nutraceuticals and nanocarriers.

Apart from the issues mentioned above, there are other challenges related to the dosage of nutraceuticals and their interactions with food, drugs, and excipients that must be considered when formulating delivery systems containing nutraceuticals. Figure 3 points out several challenges related to nutraceuticals and nanoformulations. Excessive over-the-counter use of nutraceuticals may cause lethal effects to the body, underscoring the importance of calculating the appropriate dosage based on age and health conditions. Interactions may lead to serious toxicity issues, elevated or diminished response after concomitant administration of nutraceuticals with other functional groups, drugs, or food. Moreover, the limited availability of data and documented information regarding safety, efficacy, and toxicity poses challenges when formulating dosage forms containing phytochemicals. Also, in the case of coadministration of medicine and phytochemicals, the literature does not provide enough in vitro and in vivo release and toxicity data or support evidence on drug–phytochemical interactions [401]. For example, the biocompound allicin, which has antihypertensive properties, can escalate the risk of bleeding when administered with any anticoagulant agent. It can also intensify the hypoglycemic effects of insulin. The consumption of polyphenols with stimulant medications can help mitigate tachycardia [402].

Although several merits and perks of using nanocarrier delivery systems have been documented, an incomplete picture of their toxicity profile remains an issue that requires extensive investigation. The complexation of molecules within the internal body environment raises serious safety concerns, especially in the case of long-term usage. NPs, when passing through membranes and organs, have proteins attaching to their surfaces, which can result in alterations in shape and surface charges. Hence, the NPs reaching the target area may not be the same as the ones administered. Common physical properties, such as size, shape, curvature, and free energy, influence the arrangement of proteins around the NPs. This binding with proteins may cause hindrance in enzymatic activity, unfolding of proteins, and crosslinking of thiol groups, leading to unexpected adverse outcomes [403].

The potential toxicity of NPs is a major concern because of their ability to cross cellular membranes and the blood–brain barrier. Nanocarriers with particle size ranging from 100 nm to 1000 nm can be easily taken up by phagocytes, whereas NPs smaller than 100 nm are more prone to endocytosis. NPs susceptible to phagocytosis and endocytosis need additional screening to protect healthy cells and ensure safe therapy. Although nanoformulations composed of phospholipids are claimed to be safe for body cells, extensive research is still required [404]. For example, cationic liposomes are linked to systemic toxicity, as the positive charge on the liposomal surface interacts with serum proteins and lipoproteins, which can accelerate drug aggregation and release the content even before reaching the target site. That is why high doses can cause serious harm, such as hepatic impairment and spleen toxicity. Much higher doses of lipids have been reported to cause plasma protein depletion and disturbance in the body’s hemostasis. Higher surfactant levels in NE have also been associated with interference in the tight junctions of the gastrointestinal tract and the resulting toxicity [405].

Moreover, NPs also suffer from a short half-life as they are attacked by macrophages and accumulated in the hepatic milieu. Surface coating and PEGylation are employed to facilitate the escape of NPs from the immune system. The pharmacokinetic attributes and distribution of nanomaterials are determined by their physicochemical properties, surface charge, and morphology. Nanoformulations with a particle size greater than 10 nm are primarily found in the spleen, liver, and blood, whereas those smaller than this size range can also be found in the brain, heart, lungs, and kidneys. These physicochemical attributes are sensitive to small changes during the formulation process, and they contribute to quality variations and the reproducibility of the product [406]. Therefore, the major difficulties in nanoscale product development are scaling up and ensuring the reproducibility of nanomedicines. Nanomedicines formulation procedures involve multiple steps, such as melting, homogenization, sonication, milling, emulsification, removal of organic solvents, cooling, etc., that are easier to control at a low scale. At the industrial level, controlling multiple steps with precision is far from challenging and costly. Nanomedicines produced at a large scale may have variable therapeutic effects due to a higher probability of variation and reduced reproducibility [19].

**Figure 4:**
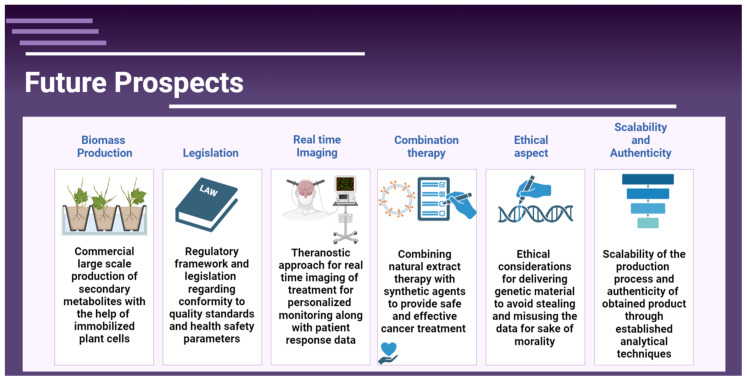
Future prospects in nanotechnology along with the nutraceutical technology sector.

A systematic evaluation and the Quality-by-Design (QbD) approach may provide a way forward to mitigate crucial variations in physicochemical properties during formulation steps and incorporate quality into the process to yield reproducible batches [407]. A summarized overview of future prospects is depicted in Figure 4.

## 7. Conclusions

LNPs built with naturally occurring lipids are one of the most reliable nanotechnologies that are being used to load bioactives. They provide a shielding effect to the cargo, ensuring its effectiveness and stability over extended periods of storage. This review article throws light on the intrinsic worth and virtues of natural compounds encapsulated in numerous LNPs. LNPs loaded with effective natural constituents provide a wide range of therapeutic options to combat and prevent ailments. Currently, the absolute necessity is to pay continuous efforts in conducting extensive in vivo experiments and clinical trials on nutraceuticals, as well as in developing a stringent regulatory framework to fully unleash the potential of nutraceutical products.

## Figures and Tables

**Table 1:** Advantages and disadvantages of preparation methods of LNPs.

Methods	Advantages	Disadvantages
Hot High-Pressure Homogenization	Large scale production, absence of organic solvent, incorporation of lipophilic and insoluble drugs	Temperature dependent degradation, intricacy of crystallization, unsuitable for heat-sensitive or hydrophilic drugs
Cold High-Pressure Homogenization	Suitable method for thermolabile compounds	Large particle size and broader size distribution
Ultrasonication	Common laboratory equipment	Wider size distribution, large amount of surfactant required, physical instability
Phase Inversion Temperature	Temperature can be reduced by surfactant incorporation, narrow size distribution, high stability	Thermolabile ingredients degradation
Precipitation	Rapid and reproducible	Toxicological issues due to solvent residue
Coacervation	Simple, avoidance of organic solvent	Applicable only to non-pH dependent drugs and alkaline nature lipids
Microemulsion	Scale up method, thermodynamically stable formulation	Multiple steps involved, diluted dispersion acquired
Double Emulsion	Applicable to hydrophilic drugs	Large particle size, low entrapment efficiency and drug loading
Emulsification	High encapsulation efficiency, low energy input, no thermal stress	Removal of organic solvent
Thin Film Hydration	MLVs are prepared, good reproducibility	Low encapsulation efficiency
Proliposome	High encapsulation efficiency, fast, simple, large quantity production	Poor reproducibility
Ether/Ethanol Injection	Simple, large quantity production, reproducible	Removal of organic solvent, poor encapsulation (in case of ethanol injection method)
Solvent Evaporation	Avoidance of heat	Solvent residue may cause toxicological problems, dilute suspension
Solvent Injection	Easy and fast, small droplets	Residual solvent, removal of organic solvent
Microfluidization	Laboratory and industrial scale method	High amount of solvent residue
Self-emulsifying system	Enhanced solubility, prevent the biodegradation of lipophilic drugs	Low drug loading capacity, drug leakage, low stability

**Table 2:** Miscellaneous phytochemicals loaded in nanocarriers with their documented potential activity between 2019–2023.

Phytochemicals	Method of Preparation	Potential Activity against	LNPs	References
Anacardic acid	Hot homogenization method	Microbial biofilm	SLN	[47]
Apigenin	Microemulsion method	Diabetic neuropathy	SLN	[48]
Baicalein	Reverse evaporation method	Acute lung injury	Liposomes	[49]
Bakuchiol	Hot homogenization method	Psoriasis	SLN	[50]
Betulinic acid	Microemulsion method	Retinal oxidative injury	SLN	[51]
Caffeic acid	Lipid film hydration	Alzheimer’s disease	Liposomes	[52]
Camphor	Emulsification method	Asthma	NE	[53]
Carvacrol	High pressure microfludization	Fungal infection	NE	[54]
Chrysin	Homogenization and sonication	Pancreatic cancer	SLN	[55]
Cinnamaldehyde	Thin film evaporation	Bacterial infection	Liposomes	[56]
Citral	High pressure homogenization	Breast cancer	NLC	[57]
Coumarin	Cold homogenization method	Drug-resistant bacterial infection	SLN	[58]
Eucalyptol	Thin film hydration	Diabetes-associated vascular endothelial injury	Liposomes	[59]
Eugenol	High speed shearing	Wound treatment	NE	[60]
Ferulic acid	Solvent evaporation	Colon cancer	SLN	[61]
Gallic acid	Double emulsion technique	Oxidation in food fortification	SLN	[62]
Hesperetin	Phase inversion temperature method	Glioblastoma	NLC	[63]
Honokiol	Low energy shaking method	Glioblastoma	NE	[64]
Hypericin	Ultrasonication method	Breast cancer	NE	[65]
Hyperoside	Thin film hydration	Hepatocellular carcinoma	Liposomes	[66]
Juglone	Thin film hydration	Cystic hydatid disease	Liposomes	[67]
Kaempferol	Emulsification method	Glioblastoma	NLC	[68]
Lapachol	Hot homogenization method	Breast cancer	NE	[69]
Licochalcone	High shear homogenization	Schistosomiasis	SLN	[70]
Lignin	High pressure homogenization	Hydrophobicity and oxidative stress	NE	[71]
Limonene	Ultrasonication method	Fungal infection (tomato preservation)	NLC	[72]
Myricetin	Ultrasonication homogenization method	Colorectal cancer	SLN	[73]
Oleanolic acid	Thin film hydration	Hepatocellular carcinoma	Liposomes	[74]
Paeoniflorin	Thin film hydration	Rheumatoid arthritis	Liposomes	[75]
Parthenolide	Thin film hydration	Cervical cancer	Liposomes	[76]
Perillyl alcohol	Hot high pressure homogenization	Brain tumor	NLC	[77]
Phloretin	Emulsification method	Inflammation	NE	[78]
Pinene	Ethanol injection method	Oxidative stress	Liposomes	[79]
Pinocembrin	Thin film hydration	Hyperglycemia	Liposomes	[80]
Proanthocyanidins	Homogenization method	Parkinson’s disease	SLN	[81]
Procyanidin	Thin film hydration	Oxidative stress	Liposomes	[82]
Pterostilbene	Ultrasonication method	Breast cancer	SLN	[83]
Pulegone	High shear homogenization	Microbial infection	SLN	[84]
Quercetin	High shear homogenization	Diabetes mellitus	NE	[85]
Thymol	Emulsification method	Oral infections	NE	[86]
Thymoquinone	Ultrasonication method	Breast cancer	NE	[87]
Umbelliferone	Thin film hydration	Dalton’s ascites lymphoma	Liposomes	[88]

**Table 3:** Use of various Cur-loaded LNPs against different types of cancers.

Cancer Type	LNPs	Particle Size (nm)	Preparation Methods	References
Lung cancer	SLN	20–80	Sol-gel	[144]
Liver cancer	NLC	90	High-pressure microfluidics	[145]
Colon cancer	SLN	<500	Coacervation	[146]
Breast cancer	SLN	152	High pressure homogenization	[147]
Breast cancer	SLN	194	High pressure homogenization	[148]
Endometrial cancer	Liposomes	100–150	Thin-film hydration	[149]
Cervical cancer	Liposome	350–390	Thin-film hydration	[150]
Hepatocellular cancer	Chitosan coated liposome	240	Thin-film hydration	[151]
Breast cancer	Surface modified liposomes	207–297	Thin-film hydration	[152]
Breast cancer	NE	199	Nanoemulsification	[153]
Lung and liver cancer	SLN	104.1	Thin-film ultrasonic hydration method	[154]
Breast cancer	SLN	175–190	Cold dilution of microemulsion	[155]
Liver cancer	NE	114.7	Emulsification method	[156]
Breast cancer	NE	40	Emulsification method	[157]
Stomach cancer	NLC	11–50	High pressure homogenization	[158]
Lung cancer	liposome	80–100	Thin-film hydration	[159]
Liver cancer	liposome	90–120	Thin-film hydration	[160]

**Table 4:** Phytochemicals possessing anti-inflammatory and antioxidative activities loaded in nanocarriers.

Phytochemicals	Formulation Technique	Excipients	Indications	Route of Delivery	References
Sulforaphane, Curcumin	hot melt emulsion	dichloromethane, stearic acid, poloxamer 188	pancreatic cancer	oral	[215]
Sulforaphane, Curcumin	hot melt emulsion	stearic acid, poloxamer 188, chitosan	pancreatic cancer	oral	[216]
Thymoquinone	high pressure homogenization	olive oil, soy lecithin, phosphatidylcholine, sorbitol, polysorbate 80, thimerosal	gastroprotective	oral	[217]
Rosmarinic acid	hot melt ultrasonication	Witepsol wax, polysorbate 80	food industry applications	oral	[218]
Rosmarinic acid	hot melt ultrasonication	carnauba wax, polysorbate 80	pharmaceutical/food industry	oral	[219]
Rosmarinic acid	hot high pressure homogenization	glycerol monostearate (GMS), polysorbate 80, soy lecithin, hydrogenated soybean phosphatidylcholine (HSPC)	Huntington’s disease	nasal	[220]
Astaxanthin	high pressure homogenization	glycerol monostearate, stearic acid, glycerol distearates, polysorbate 20,	food industry/ cosmetics	oral/topical	[221]
Astaxanthin	double emulsion solvent displacement	stearic acid, poloxamer 188, lecithin	neurological disorders	nasal	[222]
Epigallocatec-hin gallate (EGCG)	multiple emulsion (w/o/w)	soybean phosphatidylcholine, Softisan, poloxamer 188, cetyltrimethylammonium bromide (CTAB), dimethyldioctadecylammonium bromide (DDAB)	glaucoma, age-related macular degeneration	ocular	[223]
Silibinin	solvent emulsification and evaporation	tristearin, poloxamer 188, polysorbate 80	colonic diseases	oral	[224]
Lutein	high shear homogenization	glycerol stearate, Tween 80, carnauba wax, fish oil, Tween 80, poloxamer 407	food industry	oral	[225]
Lutein	high pressure homogenization	Plantacare 810, Tween 80, carnauba wax, cetyl palmitate, glyceryl tripalmitate	photoprotection	topical	[226]
Lutein	hot sonication	Tween 80 and Span 60, diethylene glycol monoethyl ether, cyclodextrin	corneal diseases	ocular	[227]
Quercetin	high pressure homogenization	caprylic/capric triglyceride, glyceryl monostearate, glycerol monolaurate, polyglyceryl-10 laurate, polyglycerol-6 monostearate, sucrose ester	functional food industries	oral	[228]
Quercetin	ultrasonication	tripalmitin, lecithin, chitosan, Tween 80	-	oral	[229]
Quercetin	emulsification and solidification	glyceryl monostearate, Tween-80. PEG 400, soy lecithin	colonic diseases	oral	[230]
Quercetin	emulsion evaporation–solidification	soy lecithin, glyceryl monostearate, stearic acid, medium-chain triglyceride, D-α-tocopheryl polyethylene glycol succinate (TPGS)	dermal infections	topical	[231]
Quercetin	hot sonication	glyceryl palmitostearate, Compritol 888, Tween 40	H_2_O_2_-induced oxidative damages	ocular	[232]
Quercetin	emulsification and solidification at low temperature	glycerol monostearate, medium-chain triglycerides, Transcutol, soy lecithin	hepatic, renal, and pulmonary disorders	oral	[233]
Luteolin	hot-microemulsion ultrasonic	glycerol monostearate, soybean lecithin, Tween 80	neurodegenerative, cancerous disorders	oral	[234]
Green tea extract	high shear homogenization	glycerol Stearate, lecithin, n-hexadecyl palmitate, Tween 80, Tween 20	bacterial infection	oral	[235]
Green tea	w/o microemulsion	polyoxyethylene stearate, poloxamer 188, glyceryl monostearate	topical application	percutaneous	[236]
Green tea extract, EGCG	thin-film rehydration	1,2-dipalmitoyl-sn-glycerol-3-phosphate-rac-(1-glycerol) (DPPG, sodium salt) and 1,2-dipalmitoyl-sn-glycero-3-phosphocholine (DPPC), cholesterol	pulmonary arterial hypertension	pulmonary	[237]
Green tea extract, EGCG	high shear homogenization and ultrasonication	Precirol ATO 5, miglyol 812, Tween 80	infectious and cancer diseases	oral	[238]
Mangiferin	homogenization and ultrasonication	Lipoid S75, polysorbate 80, tocopherol, almond oil	skin regeneration	topical	[239]
Mangiferin	homogenization and ultrasonication	Lutrol F68, miglyol 812, Compritol 888 ATO	oxidative stress-related ocular diseases	ocular	[240]
Genistein	aqueous titration	olive oil, Sefsol 218, Kolliphor RH40, PEG 200	cancer treatment	-	[241]
Chlorogenic Acid	ultrasonic emulsification	Isodecyl neopentanoate, undecyl alcohol, Caprylic/capric triglyceride, DL-alpha-tocopherol acetate, Transcutol, Labrafil, Pluronic F-68	skin infection	topical	[242]
Triptolide	microemulsion	Precirol ATO 5, Compritol 888 ATO, Geleol, Cremophor RH 40, palmitic acid, stearic acid, sodium cholate	antigen-induced arthritis	intra-articular	[243]
Triptolide	thin-film hydration	folate was conjugated with polyethylene glycol-distearoyl phosphatidylethanolamine, cholesterol	rheumatoid arthritis	intravenous injection	[244]

**Table 5:** Functional food cargo in lipid nanoparticles (SLNs, NLCs, liposomes, NEs).

Functional Food	LNPs Type	Pharmacological Activity	Limitations to Address	References
Conjugated linoleic acids	NE, NLC	anti-diabetogenic, anti-carcinogenic effects, antioxidant, and anti-inflammatory	oxidative instability	[286,287,288,289]
Buckwheat	NE	antioxidant, anti-carcinogenic, and anti-inflammatory	low oral bioavailability, poor systemic absorption	[290,291]
Grape seed polyphenols	NE, liposome	antimicrobial, anti-inflammatory, anti-carcinogenic, antiviral, and antioxidant	light and temperature instability	[292,293,294]
Probiotic lactobacilli	NE, liposome	improve the normal flora, resistance against intrusion of pathogens, including shortening of diarrhea episodes, prevention of antibiotic-related diarrhea, reduction of asthma episodes, and reduction of the incidence of some respiratory infections	processing and storage instability	[295,296,297]
β-Glucan	NE, liposome	serum cholesterol reduction, blood sugar regulation, antioxidant activity, and immunomodulatory activity	higher molecular weight, reduced solubility	[298,299]
Phytosterols and phytostanols	NE, SLN	hypocholesterolemic activity	a high melting point, waxy consistency, easy crystallization	[300,301]
Policosanol	NE, liposome	lipid-lowering/hypocholesterolemic activity, antiplatelet	limited bioavailability (5 to 12%)	[302,303]
Yellow Monascus Pigment	NE	anti-mutagenic and anti-carcinogenic properties, antimicrobial activities, potential anti-obesity activities	chemical degradation, instability	[304]
γ-Oryzanol	NE	antidiabetic, antioxidant	thermal instability and heat degradation	[305]
Monacolin K	liposome	anti-inflammatory, lipid lowering	low intestinal absorption, significant first-pass metabolism	[306]
Tocotrienol	NE, liposome	anti-proliferative, antioxidant, anti-inflammatory, anti-carcinogenic	instability and poor bioavailability	[307,308]
Soybean daidzein	NE, liposome	antioxidant, anti-inflammatory	intense metabolism and insolubility	[309,310]
Soybean genistein	SLN	neuroprotective, anti-carcinogenic	oxidation, heat, and light sensitivity, first-pass metabolism	[311]
Soybean orobol	SLN, NLC	anti-aging, antioxidant	light sensitivity, low solubility, first-pass metabolism	[312]
Soybean glycitein	NE	anti-aging, antioxidant	bitter taste, low solubility and bioavailability,	[313]
Chia mucilage	NE	texturizing agent, film-forming agent, cosmetic ingredient	physical, chemical and biological degradation, instability	[314]

**Table 6:** Herbs and spices enclosed in nanocarriers with several pharmacological activities.

Herbs/Spices	Pharmacological Activity	LNP Type	References
Tetrandrine	anti-inflammatory, immunologic, antiallergenic, antiarrhythmic	SLN	[342]
Triptolide (TP)	anti-inflammatory, immunosuppressive, antifertility, anti-neoplastic activities	SLN	[343,344]
Oridonin	anti-tumour, antibacterial, antioxidative, anti-inflammatory	SLN	[345]
Frankincense	antibacterial, antifungal, immuno-stimulating activity, anti-inflammatory,	SLN	[346]
Myrrh	antispasmodic, antiseptic, anti-tumour	SLN	[346]
Emodin	neuroprotective, anti-carcinogenic	SLN	[347]
Rosemary	hepatoprotective, antitumerogenic, antimicrobial, antioxidative	SLN, NLC	[348]
*Artemisia arborescens* L.	antiviral	SLN	[349]
Zedoary turmeric oil	antibacterial, anti-tumor, antithrombotic, hepatoprotective	NLC	[350]
Sage and Savoury	anti-diarrheal, wound healing activity, anti-inflammatory, antihypertensive	SLN	[351]
Breviscapine	antihypertensive, antithrombotic, neuroprotective	SLN	[352,353]
Neem oil	anti-inflammatory, antipyretic, antifungal, antibacterial, diuretic	SLN	[354]
Oenothera biennis Oil	antihypertensive, anti-inflammatory, sedative, astringent	SLN	[355]
Huperzine A	selective inhibitor of acetylcholinesterase	NLC	[356]
Carthamus tinctorius	antibacterial, anitoxidant, analgesic, sedative, anticonvulsant, immunosuppressive, anti-inflammatory	NLC	[357]
Zingiber Officinale	anti-inflammatory, antibacterial, hepatoprotective, anti-carcinogenic, anti-allergenic	SLN	[358]
Anethol	antifungal, antiaflatoxigenic, antioxidant	NE	[359]
Allicin	anti-inflammatory, antioxidant, neuroprotective	Liposome	[360]
Capsaicin	local anaesthetic	Liposome	[361]
17-hydroxy-jolkinolide B	anti-carcinogenic	Liposome	[362]
Kaempferol	anti-carcinogenicr, anti-inflammatory, antioxidant	NLC	[363]
Chamomile oil	antibacterial, antifungal, anti-inflammatory, antispasmodic, anti-ulcer, antiviral, sedative effect	SLN	[364]
Morin hydrate	anti-carcinogenicr	SLN	[365]
Geraniol	anti-carcinogenic	NLC	[366]
Eucalyptus oil	antibacterial, antifungal, antiseptic, antihyperglycemic, antioxidant	NLC	[367]
Gallic acid	anti-cytotoxic, antiproliferative	SLN	[368]
Linalool	antiproliferative agent	SLN, Liposome	[369,370]
Ginkgolides A and B	anti-cardiovascular, cerebrovascular	SLN	[371]
Hibiscus rosa sinensis	antidepressant, aphrodisiac, abortifacient	SLN	[372]
Amaranth oil	anti-tumor, antimicrobial, antifungal	NE	[373]
Avocado oil	hypercholesterolemia	NE	[374]
Nigella sativa	antioxidative, antimicrobial	SLN	[375]
Linseed oil	anti-inflammatory, antitumorigenic	NE, Liposome	[376,377]
Neem oil	anti-hemorrhoid, antiprotozoal, antibacterial, antitoxoplasma activity	SLN, NE	[378,379]
Glycyrrhizic acid	anti-inflammation, antiviral, detoxification, hepatic protection	modified LNP	[380]

## Data Availability

Not applicable.
